# Molecular basis for the inhibition of β-hydroxyacyl-ACP dehydratase HadAB complex from *Mycobacterium tuberculosis* by flavonoid inhibitors

**DOI:** 10.1007/s13238-015-0181-1

**Published:** 2015-06-17

**Authors:** Yu Dong, Xiaodi Qiu, Neil Shaw, Yueyang Xu, Yuna Sun, Xuemei Li, Jun Li, Zihe Rao

**Affiliations:** National Laboratory of Biomacromolecules, Institute of Biophysics, Chinese Academy of Sciences, Beijing, 100101 China; University of Chinese Academy of Sciences, Beijing, 100049 China; Structure Biology Laboratory, Tsinghua University, Beijing, 100084 China; Tianjin Key Laboratory of Protein Science, College of Life Sciences, Nankai University, Tianjin, 300071 China

**Keywords:** *Mycobacterium tuberculosis*, hotdog fold, mycolic acid, dehydratase, flavonoid, thiacetazone, isoxyl

## Abstract

Dehydration is one of the key steps in the biosynthesis of mycolic acids and is vital to the growth of *Mycobacterium tuberculosis* (*Mtb*). Consequently, stalling dehydration cures tuberculosis (TB). Clinically used anti-TB drugs like thiacetazone (TAC) and isoxyl (ISO) as well as flavonoids inhibit the enzyme activity of the β-hydroxyacyl-ACP dehydratase HadAB complex. How this inhibition is exerted, has remained an enigma for years. Here, we describe the first crystal structures of the *Mtb*HadAB complex bound with flavonoid inhibitor butein, 2’,4,4’-trihydroxychalcone or fisetin. Despite sharing no sequence identity from Blast, HadA and HadB adopt a very similar hotdog fold. HadA forms a tight dimer with HadB in which the proteins are sitting side-by-side, but are oriented anti-parallel. While HadB contributes the catalytically critical His-Asp dyad, HadA binds the fatty acid substrate in a long channel. The atypical double hotdog fold with a single active site formed by *Mtb*HadAB gives rise to a long, narrow cavity that vertically traverses the fatty acid binding channel. At the base of this cavity lies Cys61, which upon mutation to Ser confers drug-resistance in TB patients. We show that inhibitors bind in this cavity and protrude into the substrate binding channel. Thus, inhibitors of *Mtb*HadAB exert their effect by occluding substrate from the active site. The unveiling of this mechanism of inhibition paves the way for accelerating development of next generation of anti-TB drugs.

**Electronic supplementary material:**

The online version of this article (doi:10.1007/s13238-015-0181-1) contains supplementary material, which is available to authorized users.

## INTRODUCTION

Tuberculosis (TB), caused by *Mycobacterium tuberculosis* (*Mtb*), is the second most common cause of death due to a single infectious pathogen with an enormous global medical burden. In the year of 2013, approximately 30% of the world’s population were latently infected with *Mtb*, and there were nearly 9 million new TB cases and 1.5 million TB deaths (WHO 2014; Jiang et al., 2014; Zumla et al., 2013). Emergence and propagation of multidrug-resistant (MDR) and extensively drug-resistant (XDR) *Mtb* strains, and the co-infection with HIV present a huge difficulty to existing TB therapy (Comas and Gagneux, 2009; Wang et al., 2013). Amongst known pathogens, the cell wall of mycobacteria is unique primarily because of its lipid content (Goldberg et al., 2012). In particular, 2-alkyl, 3-hydroxy long-chain fatty acids known as mycolic acids (MAs) and their derivates form a dense outer sheath for shielding the inner cytoskeleton of the cell wall (Carel et al., 2014). Mycobacteria’s ability to survive and cause disease is dependent on the integrity of this lipid-rich layer referred to as myco-membrane (Bhatt et al., 2007; Takayama et al., 2005). Therefore, one approach to overpower mycobacteria is to scuttle the biosynthesis of mycolic acids (Jackson et al., 2013).

MAs issue from the Claisen condensation of an alkyl chain of medium length (C_24_–C_26_) with a long mero-mycolic chain (up to C_60_) bearing specific biochemical modifications (Asselineau et al., 2002). The biosynthetic pathway of MAs involves two types of fatty acid-synthase system (FAS): FAS-I and FAS-II. While FAS-I is a single, large polypeptide chain folded into multiple domains that harbor the catalytic sites required for the biosynthesis of fatty acids, the FAS-II is composed of a series of discrete soluble enzymes that act successively and repetitively to elongate the fatty acid chains produced by FAS-I (Sylvain Cantaloube et al., 2011). The four enzymes operating in tandem during each cycle of elongation are: 1) β-ketoacyl-ACP synthetases (KasA and KasB), 2) β-ketoacyl-ACP reductase (MabA), 3) β-hydroxyacyl-ACP dehydratases (HadAB and HadBC complexes), and 4) *trans*-2-enoyl-ACP reductase (InhA). The importance of each of these enzymatic activities in the biosynthesis of mycolic acids is well documented. Deficiency or inactivation of any enzymes above will cease the biosynthesis of MAs. Actually, these are ideal and actual targets for drug discovery (Campbell and Cronan, 2001; Marrakchi et al., 2014): the first-line anti-TB agent isoniazide (INH) and second-line agent ethionamide (ETH) act on InhA (Banerjee et al., 1994); an antibiotic thiolactomycin (TLM) inhibits the activity of KasA and KasB (Kremer et al., 2000).

**Figure 1:**
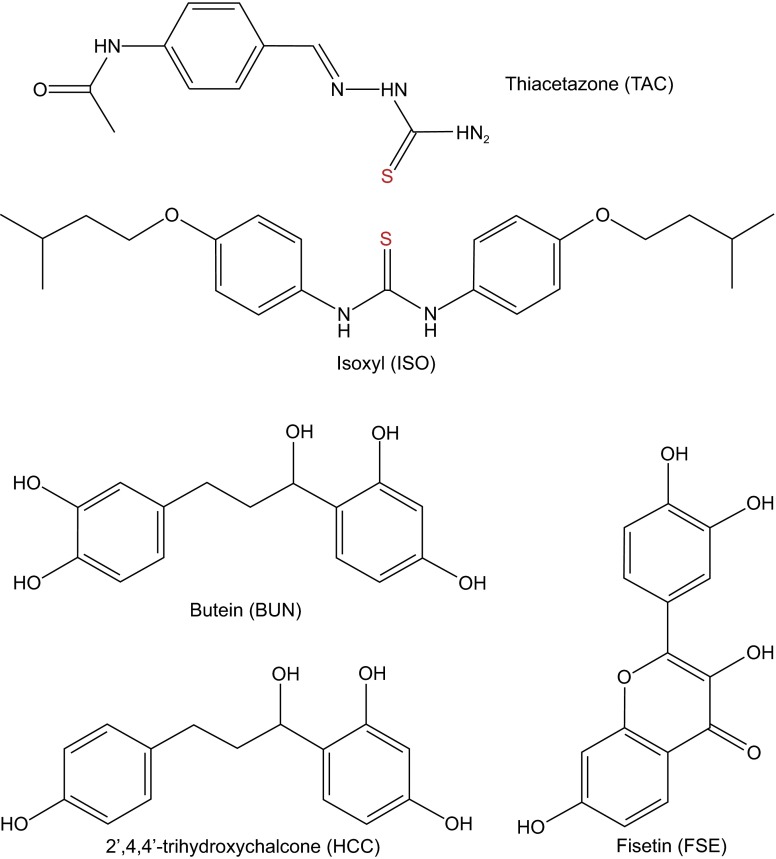
**Chemical structures of compounds of the current study related to inhibition of** ***Mtb*** **HadAB**. Thiacetazone (TAC) and isoxyl (ISO) have a sulfur containing group that could form a di-sulfide bond with Cys61 of HadA. *Mycobacterium tuberculosis* strains with C61S mutation in HadA are resistant to TAC and ISO

The β-hydroxyacyl-ACP dehydratase (Had) catalyzes the third step in the fatty acid elongation cycle by dehydrating β-hydroxyacyl-ACP to trans-2-enoyl-ACP (Fig. S1), which is the last piece to be identified in the mycobacterial FAS-II and exsits only in *Corynebacterineae* (Sacco et al., 2007). HadAB would take part, like KasA, in the early FA elongation cycles, leading to the formation of the intermediate-size (C_32_–C_42_) meromycolic chains, while HadBC, like KasB, would elongate further the intermediate-size meromycolic chains to full-size molecules (C_52_–C_64_) during the late elongation cycles (Gao et al., 2003; Sacco et al., 2007). Previously, flavonoid inhibitors targeting HadB (Rv0636) were shown to disrupt the biosynthesis of fatty acids, resulting in the depletion of the mycolic acid content of the Mycobacteria. Consequently, these flavonoids were shown to effectively inhibit the growth of *Mycobacterium bovis* BCG (Brown et al., 2007a). Besides flavonoids, two pro-drugs, isoxyl (ISO) and thiacetazone (TAC) (Fig. 1) used in the clinical treatment of tuberculosis, are also known to exert their anti-mycobacterial effect by stalling the dehydration step of the FAS-II elongation cycle (Belardinelli and Morbidoni, 2012; Coxon et al., 2013; Grzegorzewicz et al., 2012). Both these pro-drugs undergo activation by monooxygenase EthA, for unleashing their anti-mycobacterial potential (Dover et al., 2007; Kordulakova et al., 2007; Nishida and Ortiz de Montellano, 2011). How these drugs disrupt the dehydratase activity of the FAS-II system has remained an enigma for years. A lack of understanding of the molecular basis of this inhibition has been a major bottleneck in the development of next generation of drugs essential for targeting the mycolic acid component of mycobacteria.

Key to overcoming this impediment is the elucidation of the crystal structure of the *Mtb*HadAB complex that catalyzes the dehydration step. This is important also because the primary sequence of *Mtb*HadAB is very different from that of homologous enzymes like FabA and FabZ that catalyze similar reactions in other micro-organisms (Bloch, 1977; Brown et al., 2007a; Sacco et al., 2007). Currently, crystal structures of FabZ from four different sources, *Pseudomonas aeruginosa* (Protein Data Bank (PDB) code 1U1Z) (Kimber et al., 2004), *P. falciparum* (PDB code 1Z6B and 1ZHG) (Kostrewa et al., 2005; Swarnamukhi et al., 2006), *Helicobacter pylori* (PDB code 2GLL) (Zhang et al., 2008b) and *Campylobacter jejuni* (PDB code 3D6X) (Kirkpatrick et al., 2009) are available. All of them have a similar hexameric structure, displaying a classic “trimer of homodimers” organization. Because of the particular long substrate along with *Mtb*HadAB and failure to identify a specific mycobacterial homologue by BLAST searches using *E. coli* FabZ and FabA (Brown et al., 2007a), the structural insights obtained from these homologous enzymes cannot be extrapolated in its entirety to *Mtb*HadAB. Indeed, the first crystal structures of *Mtb*HadAB described here suggest the same and provide tantalizing glimpses into unique features of the active site of *Mtb*HadAB. Most importantly, the structures unveil an unexpected pocket near the active site that is targeted by flavonoids and other clinically used anti-mycobacterial drugs like TAC and ISO for inhibition of the dehydratase. Our structural study of *Mtb*HadAB provided the first three dimensional atomic models of the *Mtb*HadAB-inhibitor complexes and laid the foundation for new anti-TB drug development.

## RESULTS

### Overall structure of *Mtb*HadAB complex

The crystal structure of *Mtb*HadAB complex was solved by single wavelength anomalous dispersion (SAD) method using X-ray diffraction data collected from crystals of seleno-methionine labeled protein. The crystal belonged to space group *P*4_1_2_1_2, with unit-cell parameters *a* = *b* = 82.0 Å, *c* = 139.8 Å, *α* = *β* = *γ* = 90.0°. A Matthews coefficient of 3.56 Å^3^ Da^−1^ (Matthews, 1968; Potterton et al., 2003), corresponding to a solvent content of 65.49%, coupled with the previous biophysical identification indicated the presence of both one molecule of HadA and HadB per asymmetric unit. The final model encompassing residues 3–146 of HadA and residues 1–142 of HadB was refined to 1.75 Å resolution with an *R*_*work*_ (*R*_*free*_) value of 15.7% (18.5%) (Fig. 3A, Table S1).

**Figure 2:**
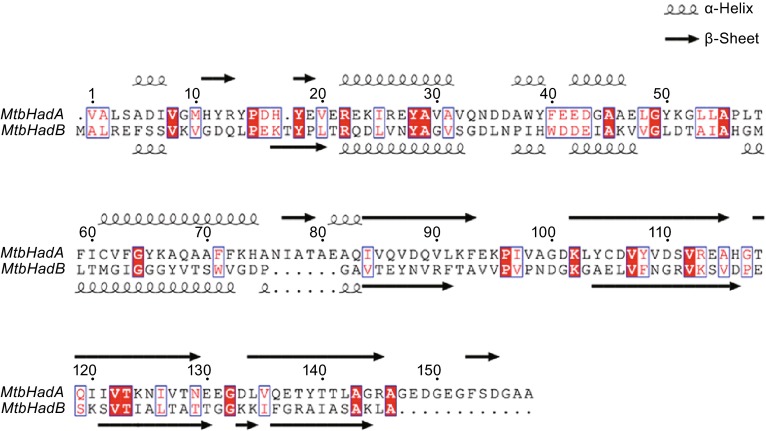
**Sequence alignment of HadA and HadB with secondary structural elements marked near the sequence**. HadA does not share any significant primary sequence identity with HadB from Blast (or only 13.57% identity calculated by ClustalW2), but their secondary structural elements distribution is similar

**Figure 3:**
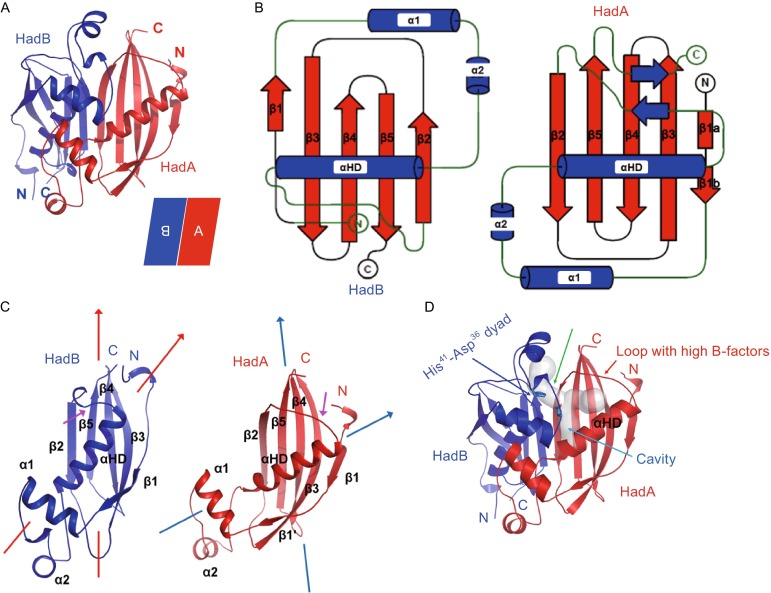
**Overall structure of** ***Mtb*** **HadAB complex**. (A) Cartoon representation of the structure of *Mtb*HadAB hetero-dimer is shown. HadA (red color) sits antiparallel to HadB (blue color). (B) Topology diagram of the structure of HadA and HadB. A short pair of strands in HadA was undefined in the native structure because of poor density. (C) Differences in positioning of αHD of HadA and HadB are shown. Direction of the axis of the β-sheet w.r.t. αHD is shown with arrows. Difference in conformation of loop connecting β2 with αHD is pointed using a magenta colored arrow. (D) Location of pockets in the active site of HadB is shown. The fatty acid binding channel and a vertical cavity traversing the fatty acid binding channel is shown in grey colored transparent surface representation. Location of catalytic dyad, His41-Asp36, is shown. Cavities were identified using CAVER software

HadA does not share any significant primary sequence identity with HadB from Blast (Fig. 2, showed by ESPript server (Robert and Gouet, 2014)). In spite of this, the overall structure of the *Mtb*HadAB complex reveals that both proteins adopt a similar hotdog fold (Fig. 3B). A central sheet composed of five strands (β1–β5) is twisted to form a concave cavity at the center. A hotdog helix, α3, (referred to as αHD hereafter) is embedded laterally in this cavity, giving the appearance of a typical hotdog fold. Helices α1 and α2 are inserted between strand β1 and αHD. Two such folds, one each contributed by HadA and HadB, sitting side-by-side, but oriented anti-parallel, result in a double hotdog fold. Thus, the overall structure of *Mtb*HadAB complex consists of a double hotdog fold (Fig. 3A).

Although the overall structures of HadA and HadB are similar, there exist several differences between the two proteins that impart distinct roles on them during catalysis (Fig. 3C and 3D). For instance, the positioning of the αHD within the concave cavity formed by the central β-sheet is different in both proteins. In HadA, the helical axis is almost perpendicular to the vertical axis of the β-strands forming the central sheet. In contrast, the αHD of HadB is placed upright, with the helical axis tilted in the direction of the vertical axis of the β-strands (Fig. 3C). This difference in the positioning of the αHD has implications for substrate binding. The orientation adopted by αHD in HadA pulls the top portion of strand β2 of HadA away from the adjacent strand β2 of HadB, creating a narrow gap between the two proteins for binding the fatty acid. Such a cavity for binding ligands is lacking in HadB. Intriguingly, electron density for a long aliphatic chain-like ligand bound by the protein during expression in *E. coli* is visible in another deep, narrow pocket that is almost perpendicular to the fatty acid binding channel (Fig. S2). Other significant differences between the structures of the two proteins include the lengths of the strands making up the central β-sheet. Notably, the length of all five β strands constituting the central sheet of HadA is longer than that of HadB. Further, the loop connecting αHD with β2 of the central sheet in HadA (residue 76–84) is longer than that in HadB (residue 75–80) (Fig. 3C). This probably facilitates formation of the substrate binding channel (Fig. 3D). Lastly, HadA is 16 residues longer at the C-terminus than HadB. These additional residues of HadA interact with the loop connecting αHD with β2 and form a pair of short anti-parallel β-strands though it was undefined in the native structure because of poor density.

### Interactions of *Mtb*HadAB hetero-tetramer and hetero-dimer

Hetero-dimerization of HadA with HadB is a prerequisite for unleashing the dehydratase activity of the complex. In agreement with this, we could not produce a stable preparation of either of the two proteins when expressed individually. A stable, hetero-oligomeric preparation of the proteins could be produced when HadA and HadB were co-expressed. Gel filtration chromatography and analytical ultra-centrifugation analysis suggested that HadA and HadB formed a hetero-tetramer in solution at 16°C (Fig. S3). This result was verified in the subsequent crystal structure of the *Mtb*HadAB complex where the two proteins formed a tight hetero-dimer in an asymmetric unit. Further analysis of symmetry mates revealed that HadA and HadB formed a face-to-face dimer of hetero-dimers (Fig. S4) similar to the dimer of double hotdog fold structure of MFE-2 hydratase 2 (Koski et al., 2005), which was consistent with our analytical ultracentrifugation and gel filtration results. Two hetero-dimer of *Mtb*HadAB was symmetry related by a two-fold axis along inside the interface which buried an area of 745 Å^2^ analyzed by PISA (Krissinel and Henrick, 2007). In this interface, helix α1 of HadB interacts with α1 and surrounding loops from the symmetry related HadB, while the N-terminal half of α1 of HadA was interacting with α1, β1b, and αHD from the symmetry related HadA.

**Figure 4:**
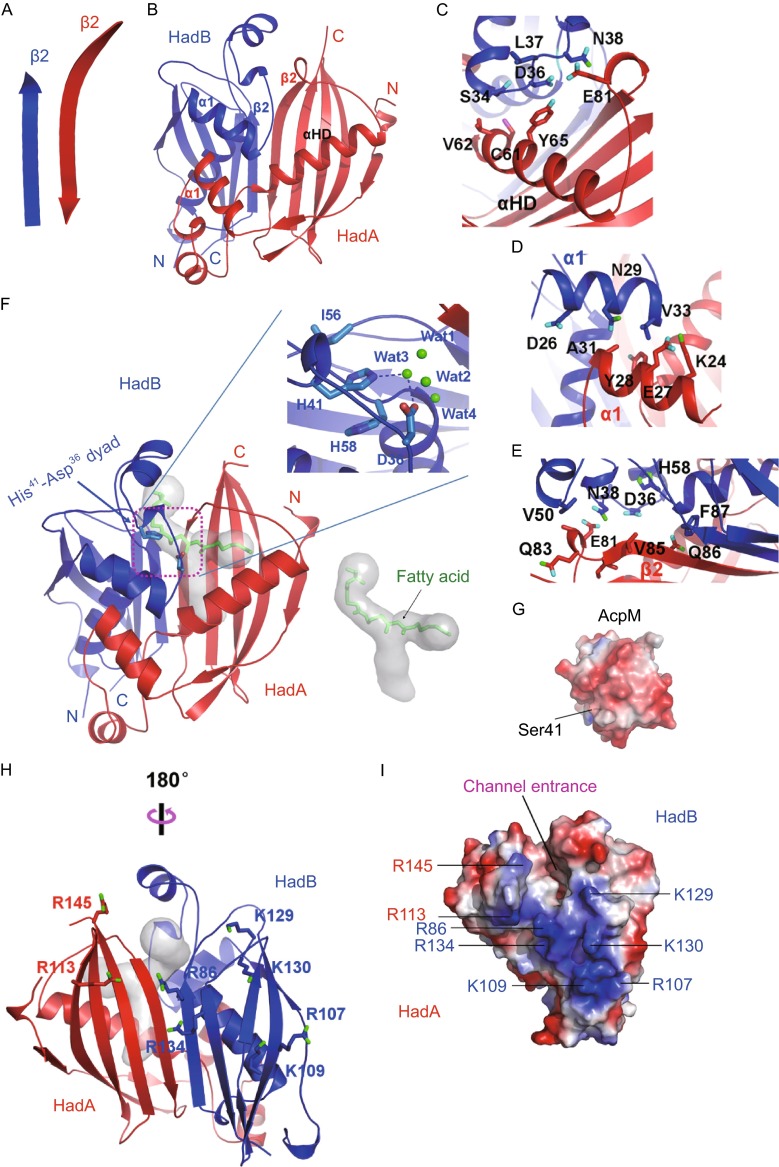
**Dimer interface and active site of** ***Mtb*** **HadAB complex**. (A) Position of β2 strands of HadA and HadB with respect to each other is shown. Main chain atoms of both these β-strands interact with each other. (B) Location of β2 strands of HadA and HadB and other structural elements involved in dimerization is shown. (C–E) Nature of intermolecular interactions between different regions of HadA and HadB is shown. Interacting residues are shown as sticks. Distances of all the interactions between HadA and HadB are listed in Table S2. (F) His-Asp catalytic dyad (shown as sticks) contributed by HadB is in vicinity of the fatty acid channel (grey surface representation). Aliphatic carbon chain of a fatty acid molecule modeled into the channel is shown on the right hand side in stick representation. Catalytic waters bonded to His-Asp dyad are shown as spheres (inset). (G) Surface electrostatic potential representation of AcpM (PDB code 1KLP) without showing the C-terminal tail. The surface is predominantly negatively charged. (H) Location of positively charged residues on the rear side of *Mtb*HadAB is shown. (I) Surface electrostatic potential representation of the view shown in panel H. Location of positively charged amino acids contributed by HadA or HadB as well as entrance of fatty acid binding channel are marked. Blue and red colors represent positive and negative potential, respectively

A close examination of the structure of the *Mtb*HadAB heterodimer reveals that hetero-dimerization is mediated by two types of inter-molecular interactions (Fig. 4). The first type of interactions involves the main chain backbone atoms of the β2 strands from both proteins. HadA and HadB interact with each other via their β2 strands such that the sheets from the two proteins are joined together to form one single contiguous sheet (Fig. 4A and 4B). The second type of inter-molecular interactions is mainly mediated by side chains (Fig. 4C–E; Tables S2 and S3) and is observed between: 1) amino acids of αHD of HadA and the helices α1 and α2, as well as the loop connecting these helices of HadB, 2) helix α1 of HadA and HadB, 3) β2 of HadA with αHD of HadB. PISA analysis revealed that hetero-dimerization of HadA with HadB resulted in burial of 1,453 Å^2^ surface area of each monomer.

### Active site of *Mtb*HadAB dehydratase

Previous bioinformatics analysis (Labonte and Townsend, 2013; Sacco et al., 2007) indicated that only HadB possesses hydratase 2 motif ([YF]-x(1,2)-[LIVG]-[STGC]-G-**D**-x-N-P-[LIV]-**H**-x(5)-[AS]) among HadA, HadB, and HadC, and the main catalytic residues Asp36 and His41 of HadB are highlighted in bold. It means that in the hetero-dimer interface of *Mtb*HadAB, only one active site was confirmed in the structure while the other site was evolutionally abolished, compared to other homodimer hotdog fold enzymes which possesses two active sites (Kimber et al., 2004; Leesong et al., 1996). The substrate of *Mtb*HadAB consists of two components—mycobacterial acyl carrier protein (AcpM) and the fatty acid (in the form of β-hydroxyacyl) (Fig. S5). The *Mtb*HadAB complex binds AcpM as well as the fatty acid during catalysis. Like other dehydratases, the fatty acid substrate binds in a narrow channel formed at the junction where HadA meets HadB (Fig. 4F). More specifically, this channel is located between αHD and the central β-sheet of HadA, a region in vicinity of helices α1 and α2 of HadB. Amino acids like Tyr65, Gln68, Ala69, Phe72, Thr79, Glu81-Ala-Gln-Ile-Val-Gln86, and Leu142 from HadA are observed lining the long, narrow channel to hold the aliphatic chain of the substrate. Asp36, Asn38, Ile40, His41, His58, Gly59, and Met60 from HadB are part of the entrance to the channel. While HadA harbors most of the residues lining this fatty acid binding channel, HadB lacks a similar channel for binding the substrate. Instead, amino acids like His41 and Asp36 of HadB are in proximity of the channel, suggesting they represent the critical catalytic dyad known to drive catalysis in hydratases (Fig. 4F). Thus, the structure of *Mtb*HadAB complex explains and provides the structural basis for the roles played by each protein. HadA functions primarily to entrench and bind the fatty acid, while HadB contributes the catalytic amino acids required for the enzymatic reaction.

Extremely long chain fatty acids (chain length > C_16_) are hydrophobic in nature. Therefore, an acyl carrier protein, AcpM, (instead of the small molecule Coenzyme A) is covalently linked to the fatty acid chain to solubilize the substrate (Bhatt et al., 2007; Mdluli et al., 1998). Furthermore, AcpM is postulated to recognize enzymes belonging to the FAS II system (Trivedi et al., 2004), including *Mtb*HadAB, and deliver the fatty acid chain to the dehydratases. Recognition probably occurs via charge and shape complementarity. AcpM is a small globular protein consisting of a four helix bundle (residues 1–83) with a flexible C-terminal loop (residues 84–115) (PDB code 1KLP). While the globular head forges protein-protein interactions, the flexible tail binds fatty acids. The surface of the helical region is highly negatively charged (Fig. 4G). Coincidently, inspection of the structure of *Mtb*HadAB around the catalytic His-Asp dyad reveals a region that could complement the shape and charge of AcpM. This region comprising of arginines at positions 113(A), 145(A), 86(B), 107(B), 134(B) and lysines at positions 109(B), 129(B), 130(B) (A and B represent HadA and HadB, respectively) is located around the entrance of the fatty acid binding channel and could form salt bridges with glutamates and aspartates of AcpM (Fig. 4H and 4I) (Wong et al., 2002). In addition, the β-sheet near the entrance of the putative fatty acid binding channel is bent, forming a bow shaped pocket, which could hold the AcpM via shape complementarity. Such a mode of binding would be similar to that inferred for FabA from *Escherichia coli* (Nguyen et al., 2014) with one notable difference. While FabA forms a homodimer that has two active site pockets, *Mtb*HadAB has only one fatty acid binding channel. Therefore, only one molecule of AcpM binds *Mtb*HadAB (Fig. S6). Consistent with this, only one region of *Mtb*HadAB in vicinity of the lone fatty acid binding channel is compatible for binding AcpM. It is noteworthy that there is a loop of HadA with high B-factors above the substrate binding channel, and it is difficult for long fatty acid chain of substrate to insert from one end and pass through the channel. So we deduced that the chain of substrate was recognized and bound by lying into the channel through the opening of the unique loop, because this loop is very flexible in structure with a higher temperature factor.

### Structures of *Mtb*HadAB-flavonoid complex

**Figure 5:**
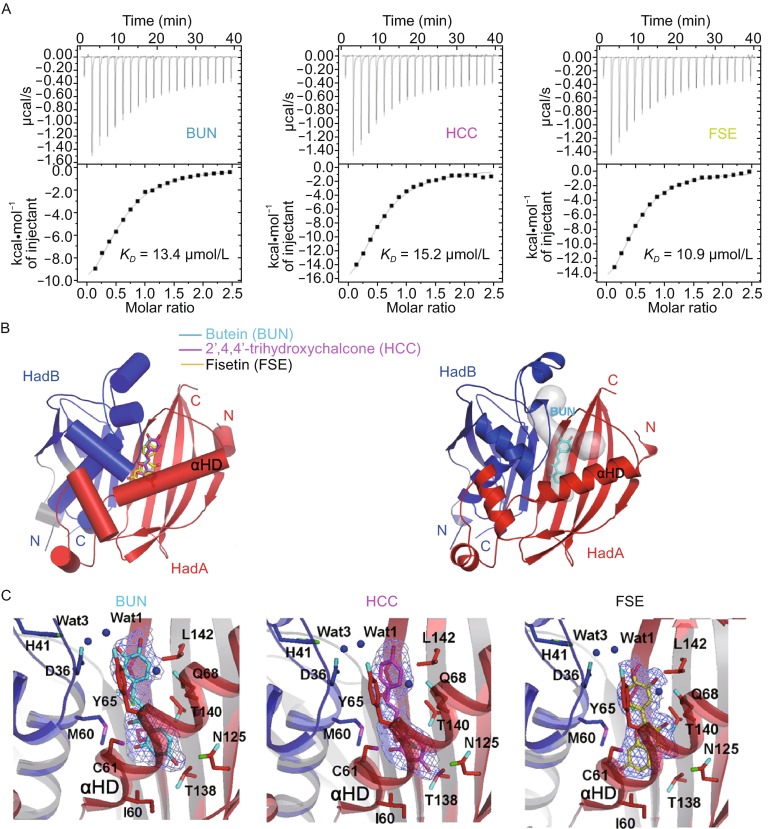
**Binding of flavonoids to** ***Mtb*** **HadAB complex**. (A) *K*_*D*_ values determined for the binding of flavonoids to *Mtb*HadAB complex by ITC. (B) Different flavonoids tested in the current study (shown as sticks) bind at the same location on *Mtb*HadAB. Upon superimposition of the structures, the ligands overlap. They bind in the cavity (right panel, gray colored surface representation) located just beneath the fatty acid binding channel. An example of butein (BUN; cyan sticks, right panel) bound in the cavity is shown. (C) Nature of amino acid interacting with flavonoids is shown. Flavonoids and amino acids are shown as sticks, while water is depicted as spheres. 2*Fo*-*Fc* electron density contoured at 1σ for the three ligands is shown

Flavonoids like butein (BUN), 2’,4,4’-trihydroxychalcone (HCC) and fisetin (FSE), are known to stall biosynthesis of mycolic acids by mycobacterial FAS-II system (Fig. 1). They have been shown to particularly inhibit the activity of HadB (Brown et al. 2007b). Consequently, these flavonoids inhibit the growth of *Mycobacterium bovis* BCG, demonstrating their utility in development of anti-TB drugs. How these flavonoids inhibit HadB is currently not understood. Results of our biophysical studies on HadA and HadB show that HadB forms a tight hetero-dimeric complex with HadA. Therefore, these flavonoids probably target the *Mtb*HadAB complex for inhibiting mycolic acid synthesis. We tested the binding of these flavonoids to the *Mtb*HadAB complex using an isothermal titration calorimetry (ITC) based assay. All the three flavonoids bound the *Mtb*HadAB complex with a *K*_*D*_ value in the 10–15 μmol/L range (Fig. 5A).

To elucidate the binding mode of these flavonoids to *Mtb*HadAB for explaining the mechanism of inhibition of HadB by flavonoids, we solved three crystal structures of the *Mtb*HadAB-flavonoid complexes by MR method using three high resolution data sets all beyond 2.2 Å. (Fig. 5B, Table S1). All the structures including native *Mtb*HadAB shared an *r.m.s.d.* not more than 0.242 Å between each other comparing corresponding Cα atom positions. Electron density for all the ligands was clear and permitted unambiguous interpretation of the mode of binding of the flavonoids (Fig. 5C). One molecule of the flavonoid binds one molecule of the *Mtb*HadAB hetero-dimer. Remarkably, structures of the complexes reveal that all the three flavonoids bind in the same deep pocket of *Mtb*HadAB that is located just beneath the substrate binding channel (Fig. 5B). Density for an aliphatic ligand was observed bound in this pocket in the native structure of *Mtb*HadAB complex (Fig. S2). Interestingly, all the atoms of the three ligands are arranged in the same vertical plane, which is perpendicular to the plane of the fatty acid binding channel. This pocket harboring the flavonoids merges with the fatty acid binding channel near the surface of the protein, in proximity of the catalytic dyad.

Specifically, the inhibitor binding pocket is located between the central β-sheet and the αHD of HadA. Considering the fact that the chemical structures of butein and 2’,4,4’-trihydroxychalcone (HCC) are almost identical; with the exception of an additional hydroxyl group on the benzene ring of butein located near the surface of the protein, it is not surprising that these two flavonoids bind *Mtb*HadAB in an almost identical manner. This observation also independently confirms the location of binding site for inhibitors. In the structures of both these binary complexes, side chains of Gln86, Gln89 of β2 and Thr138, Thr140, Leu142 of β5 stack on the rear side of the ligand along its entire length, while Ile60, Cys61, Gly64, Tyr65 of αHD and Asp36 (HadB) of the catalytic loop stack on the front side (Fig. 5C). Asn125 of β4 and Gln68 of αHD together with Met60 (HadB) from another αHD further restrain the movement of ligands. The only hydrogen bond formed by these two ligands with the protein is between the hydroxyl group of the ligand buried in the protein and side chains of either Thr138 or Asn125 (Fig. S7). A noteworthy observation is the hydrogen bonding of the additional hydroxyl group of butein with the catalytic water Wat3 bound by the His-Asp dyad. In contrast to butein and HCC, the chemical structure of fisetin is different. However, the overall mode of binding of fisetin in the pocket is similar to that of the other two ligands. Hydrogen bonds are formed between O4 and O3’ atoms of ligand and side chains of Asn125 and Gln68, respectively. In addition, O7, O3’, and O4’ atoms of the ligand are interacting with surrounding solvent molecules. Some of these waters are in proximity of the catalytic His-Asp dyad (Fig. S7).

**Figure 6:**
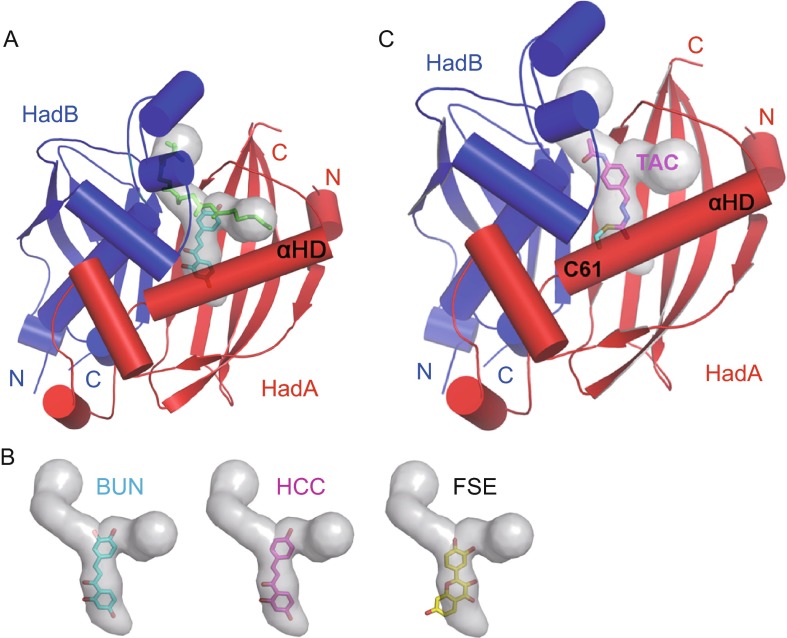
**Mechanism of inhibition of enzymatic activity of** ***Mtb*** **HadAB complex**. (A) Flavonoids bind in the cavity (gray colored surface representation) that traverses the fatty acid binding channel. An example of how binding of butein (cyan sticks) physically obstructs the placement of fatty acid (modeled as green sticks) in the substrate binding channel is shown. (B) All the three flavonoids (sticks) protrude into the substrate binding channel and perturb binding of the substrate in the active site. The cavity and substrate binding channel are shown as gray colored surface representation. (C) The inhibition model of thiacetazone (TAC, modeled as sticks) on *Mtb*HadAB. TAC is shown covalently attached to Cys61 (shown as cyan colored sticks). C61S mutation is known to confer resistance to TAC. The drug protrudes into the fatty acid binding channel (gray colored surface representation) and occludes binding of fatty acid

The molecular basis for the inhibition of *Mtb*HadAB can be envisioned based on the structures of *Mtb*HadAB bound with flavonoids. Binding of the flavonoid to *Mtb*HadAB can physically occlude binding of the fatty acid into the substrate binding channel (Fig. 6A). All the three flavonoids protrude into the fatty acid binding channel (Fig. 6B). Notably, they are in proximity of the catalytic amino acids. Therefore, the flavonoids can scuttle binding of the fatty acid or prevent it from binding in a catalytically competent orientation. Amongst the flavonoids tested for inhibition of growth of *Mycobacterium bovis* BCG strain, butein was the most effective with an MIC value of 157 μmol/L (43 μg/mL) (Brown et al., 2007b). This can be explained by examining the structures of the binary complexes of the flavonoids with *Mtb*HadAB. The O1 hydroxyl group of butein forms a hydrogen bond with the putative catalytic water Wat3 that is hydrogen bonded to both the residues of the His-Asp dyad (Figs. 5C and S7). This interaction is not observed in HCC and fisetin. Interestingly, the IC_50_ value for the inhibition of FAS II system by fisetin was 54 μg/mL, which was almost twice of that exhibited by HCC and butein. Inspection of the structures of flavonoids bound with *Mtb*HadAB reveals that the obstruction of the fatty acid binding channel by fisetin is the least efficient amongst the three flavonoids. While butein and HCC can obstruct and almost completely block the fatty acid binding channel, fisetin protrudes into the channel; but, it only partially covers the fatty acid binding channel (Fig. 6B). Thus, crystal structures of *Mtb*HadAB bound with flavonoids illustrate that butein, fisetin, and HCC exert their inhibitory effect by obstructing the placement of the fatty acid into the substrate binding site of *Mtb*HadAB.

## DISCUSSION

Inhibitors of hotdog structures have already been studied for years. A number of flavonoids and derivatives are discovered as inhibitors of β-hydroxyacyl-ACP dehydratases (Tasdemir et al., 2006). Structural studies revealed either the substrate binding site or its entrance on the dimer interface of FabZ and FabA was occupied by the inhibitors to block enzyme activity (He et al., 2009; Moynie et al., 2013; Zhang et al., 2008a, b). However, flavonoids such as butein, 2’,4,4’-trihydroxychalcone, and fisetin were not found at the substrate binding channel or the entrance on the dimer interface of *Mtb*HadAB. Instead, these small molecules were inserted into a deep pocket beneath the channel at the dimer interface. Such pocket was never found in previously solved hotdog fold structures. Part of the molecules protruded out and blocked substrate binding. These findings suggest that *Mtb*HadAB is inhibited by some flavonoids in a novel way which is different from those found in other hotdog typed dehydratases.

Combining the pivotal structural insights garnered from the crystal structures of *Mtb*HadAB in complex with flavonoids with previously known information, the molecular mechanism for inhibition of mycolic acid synthesis by TAC can be envisioned. Mycobacterial strain with C61S mutation in HadA, exhibited high level of resistance to TAC and ISO (Belardinelli and Morbidoni, 2012; Gannoun-Zaki et al., 2013; Grzegorzewicz et al., 2012). Mapping of the location of this mutation on the structure of *Mtb*HadAB reveals that the mutation is located at the N-terminal end of αHD and lies at the bottom of the flavonoid binding pocket (Fig. 6C). Conceivably, the activated forms of pro-drugs TAC and ISO are highly possible to bind at this location. Resistance of C61S mutants to TAC indicates that the only sulfur containing moiety, carbothiamide of TAC, harboring the sulfur group forms a di-sulfide bond with the sulfhydryl group of Cys61. This covalent interaction is crucial for maintaining the inhibition because modeling of the drugs into the flavonoid-binding pocket reveals that there are no other hydrogen bonds formed by the drugs with the enzyme that could help hold the drug firmly into the pocket. Once bound, inhibition of the activity of *Mtb*HadAB by TAC is then accomplished by a mechanism similar to that observed for flavonoids. The distal carbonyl group of TAC protrudes into the fatty acid binding channel of *Mtb*HadAB, occluding the binding of the substrate (Fig. 6C). This abrogates mycolic acid synthesis, which adversely affects the growth of the bacterium. Consistent with this, the covalently bound activated TAC inhibits *Mycobacterium tuberculosis* with a low MIC value of 0.25 μg/mL (Coxon et al., 2013). Addition of linear alkyl groups at this end would progressively fill up the proximal end of the fatty acid binding channel, further enhancing the inhibition exerted by TAC. In agreement with this structural observation, substitution of the R_3_ position of the aromatic ring with longer aliphatic groups has been shown to increase the inhibitory effect with a concomitant decrease in the MIC value from previous study. For instance, substitution by CH_2_CH_2_CH3, OCH_2_CH_3_, OCH_2_CH_2_CH_3_, O(CH_2_)_3_CH_3_, and OPh groups decreased the MIC 5–10 folds further. In contrast, substitution of the same position with smaller groups such as H increased the MIC from 0.25 μg/mL to 5 μg/mL (Coxon et al., 2013). Thus, inhibition by TAC can be optimized further by taking into account the nature of the binding pocket. Similar to TAC, ISO also has a sulfur containing group (Fig. 1). C61S mutation confers resistance to ISO as well (Grzegorzewicz et al., 2012). Therefore, the sulfur atom of activated ISO is also able to form a di-sulfide bond with Cys61 like TAC and bind in the flavonoid-binding pocket. In fact, both TAC and ISO could be activated first by *Mtb*EthA (Rv3854, Monooxygenase) to produce the reduced intermediates with thiol groups (Kordulakova et al., 2007; Qian and Ortiz de Montellano, 2006). And two thiol groups are able to form a di-sulfide bond. Such a mode of binding would place the bi-furcated tri-carbons at the distal end of ISO into the fatty acid binding channel, obstructing fatty acid binding. Thus, we propose that through metabolism, ISO inhibits the dehydratase activity of the *Mtb*HadAB complex using a mechanism similar to TAC and flavonoids.

The hotdog fold was first identified in the structure of *Escherichia coli* FabA (PDB code 1MKA, 1MKB) (Leesong et al., 1996) and subsequently in 4-hydroxybenzoyl-CoA thioesterase from *Pseudomonas* sp. strain CSB (PDB code 1BVQ) (Benning et al., 1998). Later on, it was recognized to be widespread in eukaryotes, bacteria, and archaea, and to be involved in a range of cellular processes, from thioester hydrolysis, to phenylacetic acid degradation and transcriptional regulation of fatty acid biosynthesis (Dillon and Bateman, 2004). Therefore, the hotdog fold is both an ancient and ubiquitous motif, with members found in the three branches of life. Both HadA and HadB were predicted to be single hotdog fold structures (Castell et al., 2005; Sacco et al., 2007). Interestingly, our structural study showed a complex double hotdog fold structure of *Mtb*hadAB hetero-dimer which is different from previously solved hotdog fold structures. The primary sequence of HadA is very different from that of HadB. In spite of this, both proteins exhibit an almost identical hotdog fold. In the crystal structure of the *Mtb*HadAB hetero-dimer, the hotdog folds of HadA and HadB sit adjacent to each other, forming a non-canonical double hotdog fold. While HadA binds the substrate, HadB contributes the catalytically critical amino acids. This distinguishes the *Mtb*HadAB double hotdog fold from that of FabA (Fig. S8). In the crystal structures of FabA from *E. coli*, two monomers of the protein are observed forming a homo-dimer that has two catalytic sites positioned in vicinity of the dimer interface. In contrast, the 2-enoyl CoA hydratase 2 (MFE2) domain of *Candida tropicalis* (PDB code 1PN4) exhibits a double hotdog fold with only one functional catalytic site (Koski et al., 2004). With regards to this, the enzyme is similar to the *Mtb*HadAB complex (Fig. S8). A Dali search (Holm and Rosenstrom, 2010) showed that the overall structure of *Mtb*HadAB is most similar to MFE-2 hydratase 2 (Koski et al., 2005). The *r.m.s.d.* between corresponding Cα positions of the two structures is 2.0 Å. However, unlike the hotdog folds of the *Mtb*HadAB complex, the two hotdog folds of MFE2 are covalently linked via a long loop traversing the two β-sheets. Moreover, the αHD helix of the MFE2 dehydratase domain that is embedded laterally in the central β-sheet is highly distorted. There is also a fungal FAS dehydratase domain that depicts the type of the triple hotdog fold (Jenni et al., 2007; Leibundgut et al., 2007). Therefore, the non-canonical double hotdog fold exhibited by the *Mtb*HadAB complex is different from known assemblies of hotdog folds. More importantly, the unique configuration of the double hotdog fold adopted by *Mtb*HadAB results in formation of a deep pocket that vertically traverses the fatty acid binding channel. The significance of this finding for the development of new anti-TB drugs can be surmised from the structural observation that flavonoids and possibly TAC and ISO bind in this pocket, which disrupts binding of the substrate. This scuttles biosynthesis of mycolic acids and forms the basis for the anti-mycobacterial effect exerted by these compounds. The insights gained from the structures of the *Mtb*HadAB complex thus open up new avenues for accelerating development of next generation of clinically useful drugs for the treatment of all types of TB.

## MATERIALS AND METHODS

### Cloning

*Rv0635* gene (encoding HadA) was amplified from *M. tuberculosis* H37Rv genomic DNA by polymerase chain reaction (PCR) using forward primer 5′-TTCCATATGGTGGCGTTGAGCGCAGACAT-3′ and reverse primer 5′-CCGCTCGAGTCACGCAGCGCCATCAGAAA-3′. *Nde*I and *Xho*I sites are underlined. The amplified gene was sub-cloned into the pRSFDuet-1 vector (Novagen). *Rv0636* gene (encoding HadB) from the same source was PCR-amplified using primers 5′-CGCGGATCCATGGCGCTGCGTGAGTTCAG-3′ and 5′-CCGCTCGAGCTACGCTAACTTCGCCGAGG-3′. *Bam*HI and *Xho*I sites are underlined. After restriction digestion, the amplified product was ligated into the pGEX-6p-1 vector (GE Healthcare). Recombinant plasmids pRSFDuet-1-HadA and pGEX-6p-1-HadB were co-transformed into *E. coli* BL21 (DE3) for expression of the *Mtb*HadAB complex. Clones were verified by DNA sequencing prior to expression studies.

### Production of *Mtb*HadAB complex

For expression of GST-tagged *Mtb*HadAB complex, cells were grown in Terrific Broth medium supplemented with 100 μg/mL ampicilin and 50 μg/mL kanamycin at 37°C. When OD_600_ reached 0.8, the culture was induced with 0.3 mmol/L isopropyl-β-D-thiogalactopyranoside (IPTG) and the culture was further incubated at 16°C for 20 h. Cells were harvested by centrifugation at 4,200 ×*g* for 30 min and re-suspended in PBS, pH 7.4. After High voltage breaker (Avestin) treatment, the lysate was centrifuged at 30,700 ×*g* for 30 min at 4°C to remove cell debris. The supernatant was loaded onto a column containing Glutathione Sepharose 4B resin (GE Healthcare) pre-equilibrated with PBS, pH 7.4. The column was washed with buffer A (50 mmol/L Tris-HCl, pH 8.0, 300 mmol/L NaCl, and 10% (*v*/*v*) glycerol) to remove non-specifically bound proteins. To remove the GST-tag linked to the N-terminus of HadB, PreScission protease (GE Healthcare) was added and the mixture was incubated overnight at 4°C. The flow through containing tag-less *Mtb*HadAB complex was collected, exchanged in buffer containing 50 mmol/L Tris-HCl, pH 8.0, 50 mmol/L NaCl, and 10% (*v*/*v*) glycerol, and applied to a Resource Q anion exchange column (GE Healthcare) equilibrated with the same buffer. *Mtb*HadAB complex bound to the column was eluted with a 0.05–1 mol/L linear gradient of NaCl. Fractions containing *Mtb*HadAB were pooled, concentrated and further purified on a Superdex 75 16/200 gel filtration column (GE Healthcare) equilibrated with buffer containing 50 mmol/L Tris-HCl, pH 8.0, 150 mmol/L NaCl, and 10% (*v*/*v*) glycerol. Pure *Mtb*HadAB eluted in a single peak. The protein was concentrated to 6 mg/mL for crystallization. SeMet labeled *Mtb*HadAB was expressed in methionine-free M9 media supplemented with 60 mg/L L-SeMet. Labeled *Mtb*HadAB was purified using the same protocol as native protein. The final yield of native and SeMet labeled protein complex was 1.5 mg and 1 mg per litre culture, respectively.

### Crystallization and structure determination

Crystallization screening experiments were first carried out at 16°C using vapor diffusion method. Drops were dispensed in 96-well plates by a Mosquito liquid handling system (TTP Labtech). Each crystallization drop contained 200 nL of *Mtb*HadAB protein (6 mg/mL) mixed with 200 nL of reservoir solution. Commercially available sparse matrix screens were used for screening crystallization conditions. Crystals grew in a solution containing 100 mmol/L HEPES, pH 7.5, 26% (*w*/*v*) PEG4000. The flavonoid inhibitors were co-crystallized with *Mtb*HadAB in the same condition. The SeMet- *Mtb*HadAB derivative crystals appeared in a different reservoir solution containing 100 mmol/L Bis-Tris, pH 6.5, 25% (*w*/*v*) PEG3350. All the crystals were optimized by varying the concentration of precipitant. Diffraction data were collected at 100 K on beamlines BL1A, BL5A, and BL17A at KEK (Tsukuba, Japan) after crystals were soaked in the reservoir solutions supplemented with 20% (*v*/*v*) glycerol for 10 s and flash frozen in liquid nitrogen. HKL2000 software (Otwinowski and Minor, 1997) was used for processing data. The structure of *Mtb*HadAB was determined by the single-wavelength anomalous diffraction (SAD) method, using the peak data from selenium derivative. All the four expected selenium sites, one in HadA and three in HadB, were located using SHELXD program (Schneider and Sheldrick, 2002; Sheldrick, 2010), and the initial phases were calculated using PHENIX (Echols et al., 2012). The model of *Mtb*HadAB complex was auto-built and then refined using native data. Manual adjustments to the model were made using COOT (Emsley and Cowtan, 2004) and refined using PHENIX. Structures of *Mtb*HadAB bound with flavonoids were solved by molecular replacement (MR) method with Phaser program (McCoy et al., 2007) in CCP4 (Potterton et al., 2003), using the native *Mtb*HadAB structure as a search template. Data collection and refinement statistics are listed in Table S1. Structural figures were prepared using PyMol (Schrodinger, 2010).

### Analytical ultracentrifugation

Sedimentation velocity experiments were performed on a Beckman XL-I analytical ultracentrifuge at 16°C. Purified protein sample was diluted with buffer to 400 μL with an A_280_ absorption of about 0.7. Sample was loaded into a conventional double-sector quartz cell and mounted in a Beckman four-hole An-60 Ti rotor. Absorption data were collected at 60,000 rpm at a wavelength of 280 nm. Continuous mass distribution was calculated from the sedimentation velocity data using the SEDFIT software program.

### Isothermal titration calorimetric assay

The binding affinities of butein, 2’,4,4’-trihydroxychalcone and fisetin against *Mtb*HadAB were assayed using an ITC-200 microcalorimeter (MicroCal) device at 25°C. *Mtb*HadAB samples were placed in the reaction cell at a concentration of 0.025 mmol/L in buffer containing 20 mmol/L Tris-HCl, pH 8.0 and 150 mmol/L NaCl. For each binding assay, flavonoids at a concentration of 0.6 mmol/L were titrated into the *Mtb*HadAB samples. The titration consisted of an initial injection of 0.4 μL followed by 20 injections of 2 μL every 2 min. The titration data and binding plot were analyzed with program MICROCAL ORIGIN. Control plots (blank) were subtracted in every titration data.

### Accession codes

The coordinates and structure factor files for native and flavonoid-bound *Mtb*HadAB have been deposited in the PDB, under accession codes 4RLJ, 4RLT, 4RLU and 4RLW, respectively.

## Electronic supplementary material

Supplementary material 1 (PDF 1102 kb)

## References

[CR1] Asselineau C, Asselineau J, Lanéelle G, Lanéelle MA (2002). The biosynthesis of mycolic acids by Mycobacteria: current and alternative hypotheses. Prog Lipid Res.

[CR2] Banerjee A, Dubnau E, Quemard A, Balasubramanian V, Um KS, Wilson T, Collins D, de Lisle G, Jacobs WR (1994). inhA, a gene encoding a target for isoniazid and ethionamide in *Mycobacterium tuberculosis*. Science.

[CR3] Belardinelli JM, Morbidoni HR (2012). Mutations in the essential FAS II beta-hydroxyacyl ACP dehydratase complex confer resistance to thiacetazone in *Mycobacterium tuberculosis* and *Mycobacterium kansasii*. Mol Microbiol.

[CR4] Benning MM, Wesenberg G, Liu R, Taylor KL, Dunaway-Mariano D, Holden HM (1998). The three-dimensional structure of 4-hydroxybenzoyl-CoA thioesterase from *Pseudomonas* sp. Strain CBS-3. J Biol Chem.

[CR5] Bhatt A, Molle V, Besra GS, Jacobs WR, Kremer L (2007). The Mycobacterium tuberculosis FAS-II condensing enzymes: their role in mycolic acid biosynthesis, acid-fastness, pathogenesis and in future drug development. Mol Microbiol.

[CR6] Bloch K (1977). Control mechanisms for fatty acid synthesis in *Mycobacterium smegmatis*. Adv Enzymol Relat Areas Mol Biol.

[CR7] Brown AK, Bhatt A, Singh A, Saparia E, Evans AF, Besra GS (2007). Identification of the dehydratase component of the mycobacterial mycolic acid-synthesizing fatty acid synthase-II complex. Microbiology.

[CR8] Brown AK, Papaemmanouil A, Bhowruth V, Bhatt A, Dover LG, Besra GS (2007). Flavonoid inhibitors as novel antimycobacterial agents targeting Rv0636, a putative dehydratase enzyme involved in *Mycobacterium tuberculosis* fatty acid synthase II. Microbiology.

[CR9] Campbell JW, Cronan JE (2001). Bacterial fatty acid biosynthesis: targets for antibacterial drug discovery. Annu Rev Microbiol.

[CR10] Cantaloube S, Veyron-Churlet R, Haddache N, Daffé M, Zerbib D (2011). The *Mycobacterium tuberculosis* FAS-II dehydratases and methyltransferases define the specificity of the mycolic acid elongation complexes. PLoS One.

[CR11] Carel C, Nukdee K, Cantaloube S, Bonne M, Diagne CT, Laval F, Zerbib D (2014). *Mycobacterium tuberculosis* proteins involved in mycolic acid synthesis and transport localize dynamically to the old growing pole and septum. PLoS One.

[CR12] Castell A, Johansson P, Unge T, Jones TA, Backbro K (2005). Rv0216, a conserved hypothetical protein from *Mycobacterium tuberculosis* that is essential for bacterial survival during infection, has a double hotdog fold. Protein Sci.

[CR13] Comas I, Gagneux S (2009). The past and future of tuberculosis research. PLoS Pathog.

[CR14] Coxon GD, Craig D, Corrales RM, Vialla E, Gannoun-Zaki L, Kremer L (2013). Synthesis, antitubercular activity and mechanism of resistance of highly effective thiacetazone analogues. PLoS One.

[CR15] Dillon SC, Bateman A (2004). The Hotdog fold: wrapping up a superfamily of thioesterases and dehydratases. BMC Bioinform.

[CR16] Dover LG, Alahari A, Gratraud P, Gomes JM, Bhowruth V, Reynolds RC, Besra GS, Kremer L (2007). EthA, a common activator of thiocarbamide-containing drugs acting on different mycobacterial targets. Antimicrob Agents Chemother.

[CR17] Echols N, Grosse-Kunstleve RW, Afonine PV, Bunkoczi G, Chen VB, Headd JJ, McCoy AJ, Moriarty NW, Read RJ, Richardson DC (2012). Graphical tools for macromolecular crystallography in PHENIX. J Appl Crystallogr.

[CR18] Emsley P, Cowtan K (2004). Coot: model-building tools for molecular graphics. Acta Crystallogr D Biol Crystallogr.

[CR19] Gannoun-Zaki L, Alibaud L, Kremer L (2013). Point mutations within the fatty acid synthase type II dehydratase components HadA or HadC contribute to isoxyl resistance in *Mycobacterium tuberculosis*. Antimicrob Agents Chemother.

[CR20] Gao LY, Laval F, Lawson EH, Groger RK, Woodruff A, Morisaki JH, Cox JS, Daffe M, Brown EJ (2003). Requirement for kasB in Mycobacterium mycolic acid biosynthesis, cell wall impermeability and intracellular survival: implications for therapy. Mol Microbiol.

[CR21] Goldberg DE, Siliciano RF, Jacobs WR (2012). Outwitting evolution: fighting drug-resistant TB, malaria, and HIV. Cell.

[CR22] Grzegorzewicz AE, Kordulakova J, Jones V, Born SE, Belardinelli JM, Vaquie A, Gundi VA, Madacki J, Slama N, Laval F (2012). A common mechanism of inhibition of the *Mycobacterium tuberculosis* mycolic acid biosynthetic pathway by isoxyl and thiacetazone. J Biol Chem.

[CR23] He L, Zhang L, Liu X, Li X, Zheng M, Li H, Yu K, Chen K, Shen X, Jiang H (2009). Discovering potent inhibitors against the beta-hydroxyacyl-acyl carrier protein dehydratase (FabZ) of *Helicobacter pylori*: structure-based design, synthesis, bioassay, and crystal structure determination. J Med Chem.

[CR24] Holm L, Rosenstrom P (2010). Dali server: conservation mapping in 3D. Nucleic Acids Res.

[CR25] Jackson M, McNeil MR, Brennan PJ (2013). Progress in targeting cell envelope biogenesis in *Mycobacterium tuberculosis*. Future Microbiol.

[CR26] Jenni S, Leibundgut M, Boehringer D, Frick C, Mikolasek B, Ban N (2007). Structure of fungal fatty acid synthase and implications for iterative substrate shuttling. Science.

[CR27] Jiang D, Zhang Q, Zheng Q, Zhou H, Jin J, Zhou W, Bartlam M, Rao Z (2014). Structural analysis of *Mycobacterium tuberculosis* ATP-binding cassette transporter subunit UgpB reveals specificity for glycerophosphocholine. FEBS J.

[CR28] Kimber MS, Martin F, Lu Y, Houston S, Vedadi M, Dharamsi A, Fiebig KM, Schmid M, Rock CO (2004). The structure of (3R)-hydroxyacyl-acyl carrier protein dehydratase (FabZ) from *Pseudomonas aeruginosa*. J Biol Chem.

[CR29] Kirkpatrick AS, Yokoyama T, Choi KJ, Yeo HJ (2009). *Campylobacter jejuni* fatty acid synthase II: structural and functional analysis of beta-hydroxyacyl-ACP dehydratase (FabZ). Biochem Biophys Res Commun.

[CR30] Kordulakova J, Janin YL, Liav A, Barilone N, Dos Vultos T, Rauzier J, Brennan PJ, Gicquel B, Jackson M (2007). Isoxyl activation is required for bacteriostatic activity against *Mycobacterium tuberculosis*. Antimicrob Agents Chemother.

[CR31] Koski MK, Haapalainen AM, Hiltunen JK, Glumoff T (2004). A two-domain structure of one subunit explains unique features of eukaryotic hydratase 2. J Biol Chem.

[CR32] Koski KM, Haapalainen AM, Hiltunen JK, Glumoff T (2005). Crystal structure of 2-enoyl-CoA hydratase 2 from human peroxisomal multifunctional enzyme type 2. J Mol Biol.

[CR33] Kostrewa D, Winkler FK, Folkers G, Scapozza L, Perozzo R (2005). The crystal structure of PfFabZ, the unique beta-hydroxyacyl-ACP dehydratase involved in fatty acid biosynthesis of *Plasmodium falciparum*. Protein Sci.

[CR34] Kremer L, Douglas JD, Baulard AR, Morehouse C, Guy MR, Alland D, Dover LG, Lakey JH, Jacobs WR, Brennan PJ (2000). Thiolactomycin and related analogues as novel anti-mycobacterial agents targeting KasA and KasB condensing enzymes in *Mycobacterium tuberculosis*. J Biol Chem.

[CR35] Krissinel E, Henrick K (2007). Inference of macromolecular assemblies from crystalline state. J Mol Biol.

[CR36] Labonte JW, Townsend CA (2013). Active site comparisons and catalytic mechanisms of the hot dog superfamily. Chem Rev.

[CR37] Leesong M, Henderson BS, Gillig JR, Schwab JM, Smith JL (1996). Structure of a dehydratase-isomerase from the bacterial pathway for biosynthesis of unsaturated fatty acids: two catalytic activities in one active site. Structure.

[CR38] Leibundgut M, Jenni S, Frick C, Ban N (2007). Structural basis for substrate delivery by acyl carrier protein in the yeast fatty acid synthase. Science.

[CR39] Marrakchi H, Laneelle MA, Daffe M (2014). Mycolic acids: structures, biosynthesis, and beyond. Chem Biol.

[CR40] Matthews BW (1968). Solvent content of protein crystals. J Mol Biol.

[CR41] McCoy AJ, Grosse-Kunstleve RW, Adams PD, Winn MD, Storoni LC, Read RJ (2007). Phaser crystallographic software. J Appl Crystallogr.

[CR42] Mdluli K, Slayden RA, Zhu Y, Ramaswamy S, Pan X, Mead D, Crane DD, Musser JM, Barry CE (1998). Inhibition of a *Mycobacterium tuberculosis* beta-ketoacyl ACP synthase by isoniazid. Science.

[CR43] Moynie L, Leckie SM, McMahon SA, Duthie FG, Koehnke A, Taylor JW, Alphey MS, Brenk R, Smith AD, Naismith JH (2013). Structural insights into the mechanism and inhibition of the beta-hydroxydecanoyl-acyl carrier protein dehydratase from *Pseudomonas aeruginosa*. J Mol Biol.

[CR44] Nguyen C, Haushalter RW, Lee DJ, Markwick PR, Bruegger J, Caldara-Festin G, Finzel K, Jackson DR, Ishikawa F, O’Dowd B (2014). Trapping the dynamic acyl carrier protein in fatty acid biosynthesis. Nature.

[CR45] Nishida CR, Ortiz de Montellano PR (2011). Bioactivation of antituberculosis thioamide and thiourea prodrugs by bacterial and mammalian flavin monooxygenases. Chem Biol Interact.

[CR46] Otwinowski Z, Minor W (1997). Processing of X-ray diffraction data collected in oscillation mode.

[CR47] Potterton E, Briggs P, Turkenburg M, Dodson E (2003). A graphical user interface to the CCP4 program suite. Acta Crystallogr D Biol Crystallogr.

[CR48] Qian L, Ortiz de Montellano PR (2006). Oxidative activation of thiacetazone by the *Mycobacterium tuberculosis* flavin monooxygenase EtaA and human FMO1 and FMO3. Chem Res Toxicol.

[CR49] Robert X, Gouet P (2014). Deciphering key features in protein structures with the new ENDscript server. Nucleic Acids Res.

[CR50] Sacco E, Covarrubias AS, O’Hare HM, Carroll P, Eynard N, Jones TA, Parish T, Daffe M, Backbro K, Quemard A (2007). The missing piece of the type II fatty acid synthase system from *Mycobacterium tuberculosis*. Proc Natl Acad Sci USA.

[CR51] Schneider TR, Sheldrick GM (2002). Substructure solution with SHELXD. Acta Crystallogr D Biol Crystallogr.

[CR52] Schrodinger, LLC (2010). The PyMOL Molecular Graphics System, Version 1.3r1.

[CR53] Sheldrick GM (2010). Experimental phasing with SHELXC/D/E: combining chain tracing with density modification. Acta Crystallogr D Biol Crystallogr.

[CR54] Swarnamukhi PL, Sharma SK, Bajaj P, Surolia N, Surolia A, Suguna K (2006). Crystal structure of dimeric FabZ of *Plasmodium falciparum* reveals conformational switching to active hexamers by peptide flips. FEBS Lett.

[CR55] Takayama K, Wang C, Besra GS (2005). Pathway to synthesis and processing of mycolic acids in *Mycobacterium tuberculosis*. Clin Microbiol Rev.

[CR56] Tasdemir D, Lack G, Brun R, Ruedi P, Scapozza L, Perozzo R (2006). Inhibition of *Plasmodium falciparum* fatty acid biosynthesis: evaluation of FabG, FabZ, and FabI as drug targets for flavonoids. J Med Chem.

[CR57] Trivedi OA, Arora P, Sridharan V, Tickoo R, Mohanty D, Gokhale RS (2004). Enzymic activation and transfer of fatty acids as acyl-adenylates in mycobacteria. Nature.

[CR58] Wang L, Li J, Wang X, Liu W, Zhang XC, Li X, Rao Z (2013). Structure analysis of the extracellular domain reveals disulfide bond forming-protein properties of *Mycobacterium tuberculosis* Rv2969c. Protein Cell.

[CR59] WHO (2014) Global tuberculosis report 2014 (Geneva, World Health Organization)

[CR60] Wong HC, Liu G, Zhang YM, Rock CO, Zheng J (2002). The solution structure of acyl carrier protein from *Mycobacterium tuberculosis*. J Biol Chem.

[CR61] Zhang L, Kong Y, Wu D, Zhang H, Wu J, Chen J, Ding J, Hu L, Jiang H, Shen X (2008). Three flavonoids targeting the beta-hydroxyacyl-acyl carrier protein dehydratase from *Helicobacter pylori*: crystal structure characterization with enzymatic inhibition assay. Protein Sci.

[CR62] Zhang L, Liu W, Hu T, Du L, Luo C, Chen K, Shen X, Jiang H (2008). Structural basis for catalytic and inhibitory mechanisms of beta-hydroxyacyl-acyl carrier protein dehydratase (FabZ). J Biol Chem.

[CR63] Zumla A, George A, Sharma V, Herbert N, Masham Baroness, Ilton BM (2013). WHO’s 2013 global report on tuberculosis: successes, threats, and opportunities. Lancet.

