# Supplementary material for: Molecular basis for the inhibition of β-hydroxyacyl-ACP dehydratase HadAB complex from Mycobacterium tuberculosis by flavonoid inhibitors
Source: Protein Cell. 2015 Jun 17;6(7):504–17. doi: 10.1007/s13238-015-0181-1 (PMC4491049; doi:10.1007/s13238-015-0181-1)
Supplement: Supplementary file 1 — Supplementary material 1 (PDF 1102 kb) [file 13238_2015_181_MOESM1_ESM.pdf]

Supplementary Information

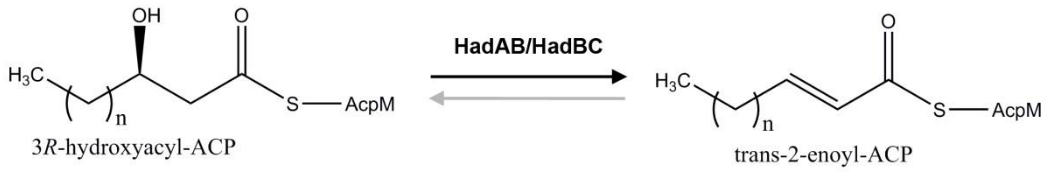

Supplementary Figure S1 The scheme of *Mtb*HadAB/*Mtb*HadBC dehydration reaction. The reaction is reversible. However, in the context of FAS-II elongation cycle, this reaction tends to the dehydration direction.

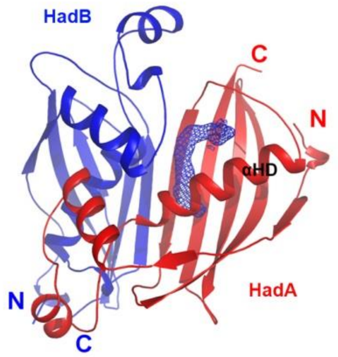

Supplementary Figure S2 Clear difference electron density (2*Fo-Fc* map for the native *Mtb*HadAB; blue colored mesh) was observed for an aliphatic ligand bound in the cavity. The ligand was bound to *Mtb*HadAB during its expression in *E. coli*.

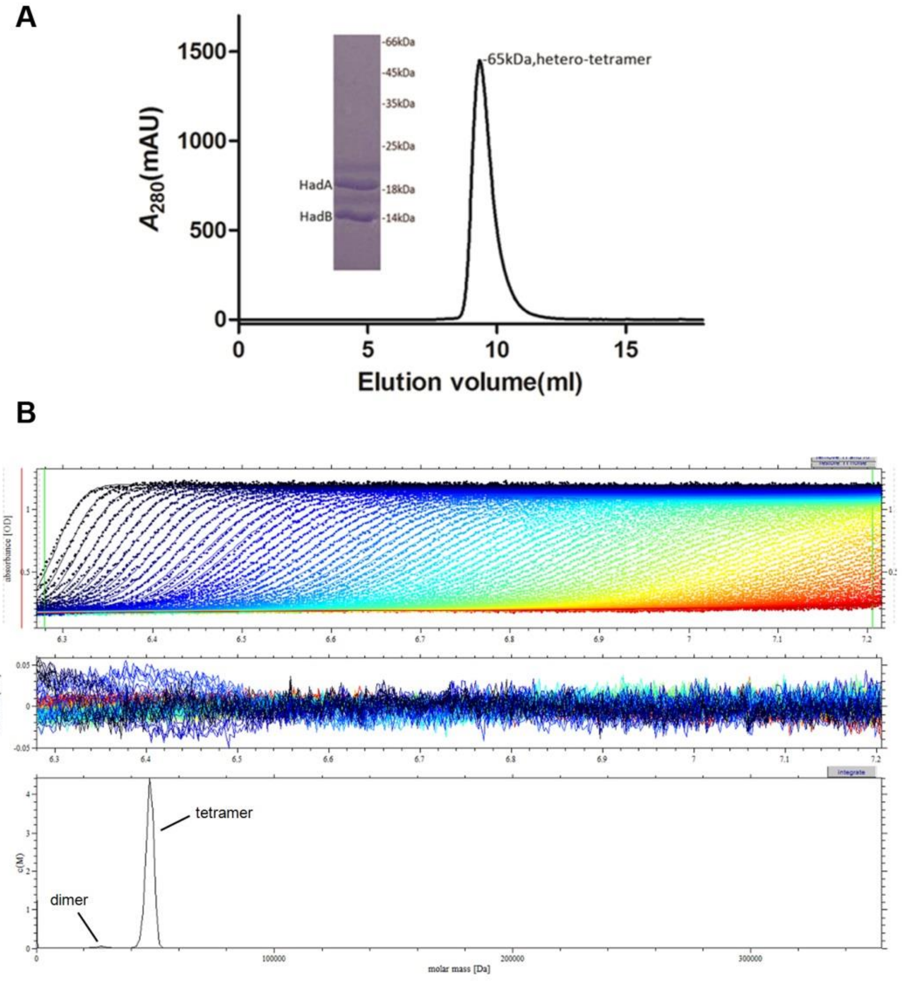

Supplementary Figure S3 Experimental analysis suggested *Mtb*HadAB complex formed a hetero-tetramer in solution at 16 ℃.

(A) Gel filtration chromatography using a Superdex 75 column and SDS-PAGE analysis (inset) of *Mtb*HadAB complex. An elution peak volume of 9.3 mL corresponded to an approximate molecular mass of 65 kDa based on calibration of the column using standard molecular weight markers.

(B) Analytical ultracentrifugation analysis of *Mtb*HadAB complex. The upper panel shows the raw data from the time-course measurement of absorbance of the sample at 280 nm along the sample cell length. The middle panel shows residuals after fitting the data to the continuous size-distribution model. The lower panel shows the continuous mass distribution for the calculated solution. *Mtb*HadAB predominantly exists as a tetramer in solution.

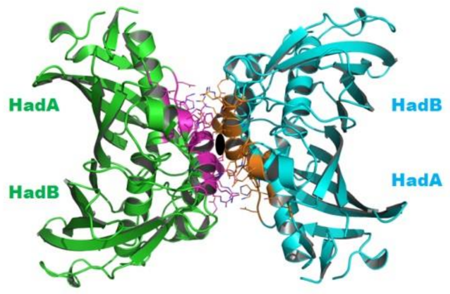

Supplementary Figure S4 Overall structure of a tetramer of *Mtb*HadAB complex and interactions between two dimers in *Mtb*HadAB tetramer. Two dimers of *Mtb*HadAB (green and cyan) are symmetric related by a two-fold axis (dark ellipse) along inside the interface between each other. Interacting residues from the two dimers are colored in magenta and orange. Side chains of these residues are shown as sticks.

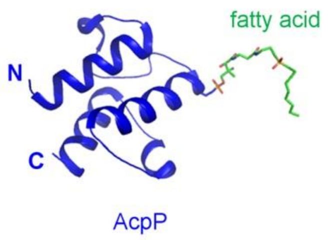

Supplementary Figure S5 Cartoon representation of AcpP bound with fatty acid. The cartoon model is shown as an example similar to substrate of *Mtb*HadAB, and it was drawn using the structure of FabA from *E. coli* in complex with acyl-carrier protein bound with fatty acid (PDB code 4KEH). FabA protein is not shown. *Mtb*AcpM delivers very long chain fatty acids to *Mtb*HadAB for catalysis.

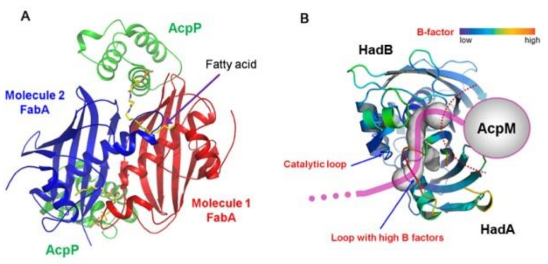

Supplementary Figure S6 Mode of binding of substrate to FAS-II dehydratases
An acyl carrier protein delivers fatty acid to dehydratases of FAS-II system. Panel (A) depicts a dimer of FabA from *E. coli* (PDB code 4KEH) in complex with two molecules of AcpP bound with fatty acid. A homodimer of FabA has two active sites. Panel (B) shows a model for binding of AcpM to the *Mtb*HadAB heterodimer. Fatty acid is depicted as a magenta ribbon, while AcpM is shown as a grey sphere binding to the bow shaped pocket (dashed red curve) at the back of *Mtb*HadAB. Substrate binding channel is shown in gray surface representation. The rainbow color depicts the level of residue temperature factor. The higher the temperature factor, the redder the color. *Mtb*HadAB has only one fatty acid binding channel.

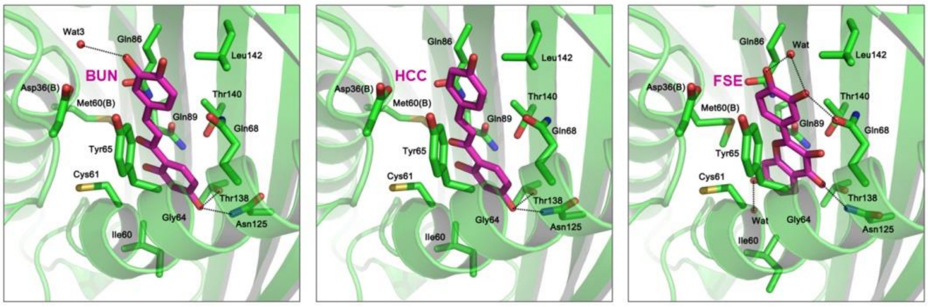

Supplementary Figure S7 Three flavonoid inhibitors bind in the pocket. Butein (BUN), 2’,4,4’-trihydroxychalcone (HCC) and Fisetin (FSE) are shown as sticks and colored in magenta. Side chains of surrounded residues are also shown as sticks. Interacting water molecules are shown as spheres. Hydrogen bond interactions are marked with dashed lines.

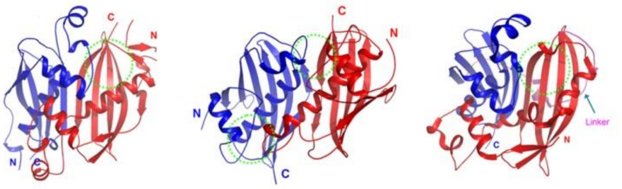

Supplementary Figure S8 Structures of homologues of *Mtb*HadAB. Although the dehydratase *Mtb*HadAB does not share any sequence identity with other dehydratases beyond *Corynebacterineae*, the overall fold adopted by the protein is similar to other dehydratases like FabA from *E. coli* (PDB code 4KEH) and MFE-2 from *Candida tropicalis* (PDB code 1PN4). While *Mtb*HadAB forms a hetero-dimer with a single active site, FabA forms a homo-dimer with two active sites. MFE-2 hydratase domain forms a pseudo-dimer; a linker connects two hotdog subunits, with a single active site for catalysis.

Supplementary Tables 1-3

Supplementary Table 1 Data collection and crystallographic statistics.

Data set	Se-Met-HadAB (peak)	HadAB (native)	HadAB-BUN	HadAB-HCC	HadAB-FSE
**Data collection**					
Space group	$P4_1212$	$P4_1212$	$P4_1212$	$P4_1212$	$P4_1212$
Unit-cell parameters					
a, b, c (Å)	a=b=82.2, c=142.3	a=b=82.0, c=139.8	a=b=82.4, c=140.8	a=b=82.4, c=141.5	a=b=82.3, c=140.2
α, β, γ (°)	α=β=γ=90.0	α=β=γ=90.0	α=β=γ=90.0	α=β=γ=90.0	α=β=γ=90.0
Wavelength (Å)	0.97905	1.00000	0.98000	1.00000	0.98000
Resolution (Å)	50.00-2.20	50.00-1.75	50.00-2.20	50.00-2.20	50.00-2.05
	(2.28-2.20)[a]	(1.81-1.75)	(2.28-2.20)	(2.28-2.20)	(2.12-2.05)
Total observation	442,538	462,997	236,291	237,827	294,370
Unique reflections	25,553	48,837	25,519	25,514	31,026
Data completeness (%)	100.0 (100.0)	99.9 (100.0)	99.9 (100.0)	99.7 (100.0)	99.8 (99.9)
R_{merge}[b]	0.100 (0.413)	0.058 (0.424)	0.084 (0.415)	0.060 (0.419)	0.084 (0.440)
$I/\delta(I)$	31.2 (8.9)	35.8 (5.8)	26.8 (5.9)	35.0 (5.5)	28.1 (5.7)
Redundancy	17.3 (18.3)	9.5 (9.6)	9.3 (9.4)	9.3 (9.6)	9.5 (9.6)
**Refinement**					
Resolution (Å)		36.68-1.75	40.78-2.20	36.66-2.20	40.62-2.05
Reflections used		48,754	25,440	25,449	30,966
R_{work} / R_{free}[c]		0.157 / 0.185	0.164 / 0.195	0.186 / 0.213	0.158 / 0.190
No. of non-H atoms					
HadA / HadB		1,170 / 1,085	1,226 / 1,080	1,214 / 1,080	1,219 / 1,080
Ligands		78	32	45	33
Water		199	173	145	245
Average B factor ($Å^2$)					
HadA / HadB		31.2 / 28.6	43.0 / 40.3	53.3 / 50.3	36.6 / 34.4
Ligands		52.4	55.3	67.2	34.5
Water		40.9	46.2	52.0	43.2
R.m.s. deviation					
Bond lengths (Å)		0.019	0.011	0.003	0.012

Bond angles (°)	1.682	1.328	0.711	1.234
Ramachandran plot (%)				
Favored	98.3	98.0	97.3	97.0
Allowed	1.7	2.0	2.7	3.0
Outliers	0.0	0.0	0.0	0.0

[a]Values in parentheses are for the highest resolution shell.

[b]$R_{merge}=\Sigma_h\Sigma_i|I_{ih}-\langle I_h\rangle|/\Sigma_h\Sigma_i\langle I_h\rangle$, where $\langle I_h\rangle$ is the mean intensity of the observations of I_{ih} of reflection h.

[c]$R_{work}=\Sigma_h|F_o-F_c|/\Sigma_h F_o$, where F_o and F_c are the observed and calculated structure factor amplitudes of reflection h.

R_{free} is mathematically equivalent to R_{work}, but was measured over 5% of the data.

Supplementary Table 2 List of intermolecular interactions (≤ 4 Å) between the subunits of *Mtb*HadAB hetero-dimer.

Interactions were calculated by NCONTACT program of CCP4, Hydrogen bonds are highlighted in green, while salt bridges are highlighted in blue.

No.	Chain A				Dist. [Å]	Chain B			
1	A:	GLU	47	[O]	3.60	B:	GLY	-1	[CA]
2	A:	GLU	47	[O]	3.40	B:	GLY	-1	[C]
3	A:	GLY	49	[N]	3.82	B:	GLY	-1	[O]
4	A:	GLU	47	[O]	3.16	B:	GLY	-1	[O]
5	A:	LEU	48	[CA]	3.30	B:	GLY	-1	[O]
6	A:	LEU	48	[C]	3.18	B:	GLY	-1	[O]
7	A:	LEU	48	[O]	3.23	B:	GLY	-1	[O]
8	A:	GLN	33	[OE1]	3.72	B:	TYR	20	[CB]
9	A:	GLN	33	[O]	3.87	B:	TYR	20	[CE2]
10	A:	GLN	33	[OE1]	3.51	B:	TYR	20	[CD2]
11	A:	GLN	33	[OE1]	3.55	B:	PRO	21	[CD]
12	A:	GLN	33	[CD]	3.66	B:	PRO	21	[O]
13	A:	GLN	33	[OE1]	3.54	B:	PRO	21	[O]
14	A:	GLN	33	[NE2]	2.98	B:	PRO	21	[O]
15	A:	GLN	33	[NE2]	3.92	B:	LEU	22	[CD2]
16	A:	ALA	31	[CA]	3.79	B:	ASN	29	[OD1]
17	A:	ALA	31	[CB]	3.49	B:	ASN	29	[OD1]
18	A:	VAL	32	[CG1]	3.65	B:	TYR	30	[CE1]
19	A:	TYR	28	[CE1]	3.61	B:	TYR	30	[CE1]
20	A:	TYR	28	[CZ]	3.99	B:	TYR	30	[CE1]
21	A:	TYR	28	[OH]	3.80	B:	TYR	30	[CE1]
22	A:	TYR	28	[OH]	3.90	B:	TYR	30	[CZ]
23	A:	CYS	61	[SG]	3.44	B:	TYR	30	[OH]

24	A:	TYR	28	[	CE1	]	3.72	B:	TYR	30	[	OH	]
25	A:	TYR	28	[	CZ	]	3.84	B:	TYR	30	[	OH	]
26	A:	TYR	28	[	OH	]	3.02	B:	TYR	30	[	OH	]
27	A:	GLU	27	[	OE2	]	3.87	B:	VAL	33	[	CA	]
28	A:	LYS	24	[	NZ	]	3.90	B:	VAL	33	[	CA	]
29	A:	GLU	27	[	OE2	]	3.84	B:	VAL	33	[	CB	]
30	A:	GLU	27	[	OE2	]	3.64	B:	VAL	33	[	CG1	]
31	A:	GLU	27	[	CG	]	3.81	B:	VAL	33	[	CG2	]
32	A:	GLU	27	[	OE2	]	3.48	B:	VAL	33	[	CG2	]
33	A:	LYS	24	[	NZ	]	3.70	B:	VAL	33	[	C	]
34	A:	LYS	24	[	NZ	]	2.77	B:	VAL	33	[	O	]
35	A:	LYS	24	[	CE	]	3.51	B:	VAL	33	[	O	]
36	A:	TYR	65	[	CE1	]	3.52	B:	GLY	35	[	O	]
37	A:	TYR	65	[	CZ	]	3.92	B:	ASP	36	[	CA	]
38	A:	TYR	65	[	OH	]	3.98	B:	ASP	36	[	CA	]
39	A:	TYR	65	[	OH	]	3.76	B:	ASP	36	[	OD1	]
40	A:	GLU	81	[	OE2	]	3.84	B:	ASN	38	[	N	]
41	A:	GLU	81	[	CD	]	3.56	B:	ASN	38	[	CA	]
42	A:	GLU	81	[	OE1	]	3.50	B:	ASN	38	[	CA	]
43	A:	GLU	81	[	OE2	]	3.71	B:	ASN	38	[	CA	]
44	A:	GLU	81	[	CG	]	3.97	B:	ASN	38	[	CB	]
45	A:	GLU	81	[	CD	]	3.76	B:	ASN	38	[	CB	]
46	A:	GLU	81	[	OE2	]	3.84	B:	ASN	38	[	CB	]
47	A:	GLU	81	[	CG	]	3.53	B:	ASN	38	[	CG	]
48	A:	GLU	81	[	CD	]	3.86	B:	ASN	38	[	CG	]
49	A:	GLU	81	[	CG	]	3.83	B:	ASN	38	[	OD1	]
50	A:	GLU	81	[	CG	]	3.56	B:	ASN	38	[	ND2	]
51	A:	GLU	81	[	O	]	3.30	B:	ASN	38	[	ND2	]
52	A:	GLU	81	[	OE1	]	3.25	B:	PRO	39	[	CD	]
53	A:	GLN	86	[	OE1	]	3.30	B:	MET	60	[	CG	]
54	A:	GLN	86	[	OE1	]	3.70	B:	MET	60	[	SD	]
55	A:	GLN	86	[	NE2	]	3.53	B:	MET	60	[	SD	]
56	A:	GLN	86	[	CD	]	4.00	B:	MET	60	[	SD	]
57	A:	THR	58	[	CB	]	3.76	B:	MET	60	[	CE	]
58	A:	THR	58	[	CG2	]	3.82	B:	MET	60	[	CE	]
59	A:	ALA	31	[	O	]	3.36	B:	LEU	61	[	CD1	]
60	A:	VAL	32	[	CG1	]	3.87	B:	LEU	61	[	CD1	]
61	A:	THR	58	[	CG2	]	3.75	B:	MET	63	[	CG	]
62	A:	GLN	89	[	CB	]	3.68	B:	MET	63	[	SD	]
63	A:	GLN	89	[	CG	]	3.90	B:	MET	63	[	SD	]
64	A:	LEU	91	[	CD1	]	3.77	B:	MET	63	[	CE	]
65	A:	GLN	89	[	O	]	3.62	B:	MET	63	[	CE	]
66	A:	THR	58	[	CG2	]	3.99	B:	MET	63	[	C	]
67	A:	THR	58	[	OG1	]	3.99	B:	GLY	64	[	N	]

68	A:	THR	58	[	CG2	]	3.84	B:	GLY	64	[	N]
69	A:	THR	58	[	OG1	]	3.26	B:	GLY	64	[	CA]
70	A:	THR	58	[	CG2	]	3.74	B:	GLY	64	[	CA]
71	A:	ASN	34	[	ND2	]	3.92	B:	GLY	64	[	C]
72	A:	VAL	32	[	CB	]	3.90	B:	GLY	64	[	C]
73	A:	ASN	34	[	ND2	]	2.99	B:	GLY	64	[	O]
74	A:	ASN	34	[	CG	]	3.75	B:	GLY	64	[	O]
75	A:	ASN	34	[	OD1	]	3.67	B:	GLY	64	[	O]
76	A:	VAL	32	[	O	]	3.88	B:	ILE	65	[	N]
77	A:	VAL	32	[	O	]	3.54	B:	ILE	65	[	CA]
78	A:	VAL	32	[	O	]	3.46	B:	ILE	65	[	CG1]
79	A:	GLN	33	[	NE2	]	3.97	B:	ILE	65	[	CD1]
80	A:	LEU	57	[	CD1	]	3.85	B:	GLY	67	[	CA]
81	A:	LEU	57	[	CD1	]	3.90	B:	GLY	67	[	O]
82	A:	ASN	34	[	ND2	]	3.43	B:	GLY	68	[	N]
83	A:	ASN	34	[	CG	]	3.74	B:	GLY	68	[	N]
84	A:	ASN	34	[	OD1	]	3.51	B:	GLY	68	[	N]
85	A:	ASN	34	[	ND2	]	3.29	B:	GLY	68	[	CA]
86	A:	ASN	34	[	CG	]	3.45	B:	GLY	68	[	CA]
87	A:	ASN	34	[	OD1	]	3.48	B:	GLY	68	[	CA]
88	A:	TYR	39	[	OH	]	3.69	B:	THR	71	[	CB]
89	A:	LEU	57	[	CD2	]	3.78	B:	THR	71	[	OG1]
90	A:	LEU	57	[	CG	]	3.37	B:	THR	71	[	OG1]
91	A:	LEU	57	[	CD1	]	3.27	B:	THR	71	[	OG1]
92	A:	TYR	39	[	OH	]	3.40	B:	THR	71	[	CG2]
93	A:	TRP	38	[	CZ2	]	3.97	B:	THR	71	[	O]
94	A:	ASP	36	[	OD2	]	3.85	B:	SER	72	[	CA]
95	A:	ASP	36	[	OD2	]	3.41	B:	SER	72	[	CB]
96	A:	ASP	36	[	OD1	]	3.90	B:	SER	72	[	CB]
97	A:	ASP	36	[	CG	]	3.66	B:	SER	72	[	OG]
98	A:	ASP	36	[	OD2	]	2.68	B:	SER	72	[	OG]
99	A:	ASP	36	[	OD1	]	3.89	B:	SER	72	[	OG]
100	A:	TRP	38	[	CH2	]	3.84	B:	GLY	75	[	CA]
101	A:	TRP	38	[	CH2	]	3.66	B:	GLY	75	[	C]
102	A:	TRP	38	[	CZ3	]	3.64	B:	GLY	75	[	O]
103	A:	TRP	38	[	CH2	]	3.68	B:	GLY	75	[	O]
104	A:	LEU	48	[	CD2	]	3.83	B:	GLY	75	[	O]
105	A:	TYR	50	[	OH	]	3.96	B:	ASP	76	[	CG]
106	A:	LEU	48	[	CD1	]	3.96	B:	ASP	76	[	OD1]
107	A:	TYR	50	[	OH	]	3.48	B:	ASP	76	[	OD1]
108	A:	TYR	50	[	CE2	]	3.82	B:	ASP	76	[	OD1]
109	A:	TYR	50	[	OH	]	3.55	B:	ASP	76	[	OD2]
110	A:	TRP	38	[	CD1	]	3.54	B:	PRO	77	[	CG]
111	A:	TRP	38	[	NE1	]	3.38	B:	PRO	77	[	CG]

112	A:	LEU	54	[	CD1	]	3.94	B:	PRO	77	[	CG	]
113	A:	TYR	39	[	CE1	]	3.86	B:	PRO	77	[	CG	]
114	A:	TYR	39	[	OH	]	3.69	B:	PRO	77	[	CG	]
115	A:	TRP	38	[	CD1	]	3.89	B:	PRO	77	[	CD	]
116	A:	TRP	38	[	NE1	]	3.57	B:	PRO	77	[	CD	]
117	A:	TRP	38	[	CE2	]	3.66	B:	PRO	77	[	CD	]
118	A:	LEU	54	[	CD1	]	4.00	B:	PRO	77	[	CD	]
119	A:	TYR	50	[	OH	]	3.96	B:	GLY	78	[	N	]
120	A:	TYR	50	[	OH	]	3.31	B:	GLY	78	[	CA	]
121	A:	PRO	96	[	CB	]	3.92	B:	GLY	78	[	CA	]
122	A:	TYR	50	[	OH	]	3.92	B:	GLY	78	[	C	]
123	A:	LEU	57	[	CD1	]	3.90	B:	VAL	80	[	CG1	]
124	A:	PHE	93	[	CE2	]	3.81	B:	VAL	80	[	CG1	]
125	A:	PHE	93	[	CD2	]	3.93	B:	VAL	80	[	CG1	]
126	A:	PHE	93	[	CD2	]	4.00	B:	VAL	80	[	C	]
127	A:	PHE	93	[	CB	]	3.55	B:	VAL	80	[	O	]
128	A:	PHE	93	[	CG	]	3.90	B:	VAL	80	[	O	]
129	A:	PHE	93	[	CD2	]	3.54	B:	VAL	80	[	O	]
130	A:	PHE	93	[	N	]	3.85	B:	THR	81	[	C	]
131	A:	PHE	93	[	CD2	]	3.65	B:	THR	81	[	C	]
132	A:	LYS	92	[	CA	]	3.73	B:	THR	81	[	O	]
133	A:	LYS	92	[	C	]	3.79	B:	THR	81	[	O	]
134	A:	PHE	93	[	N	]	2.94	B:	THR	81	[	O	]
135	A:	PHE	93	[	CA	]	3.85	B:	THR	81	[	O	]
136	A:	PHE	93	[	CB	]	3.97	B:	THR	81	[	O	]
137	A:	PHE	93	[	CD2	]	3.83	B:	THR	81	[	O	]
138	A:	PHE	93	[	O	]	3.61	B:	THR	81	[	O	]
139	A:	PHE	93	[	CD2	]	3.67	B:	GLU	82	[	N	]
140	A:	PHE	93	[	CE2	]	3.93	B:	GLU	82	[	CA	]
141	A:	PHE	93	[	CD2	]	3.76	B:	GLU	82	[	CA	]
142	A:	LEU	91	[	O	]	3.17	B:	GLU	82	[	CA	]
143	A:	LEU	91	[	O	]	3.37	B:	GLU	82	[	CB	]
144	A:	PHE	93	[	CE2	]	3.80	B:	GLU	82	[	C	]
145	A:	LEU	91	[	O	]	3.56	B:	GLU	82	[	C	]
146	A:	PHE	93	[	CE2	]	3.86	B:	TYR	83	[	N	]
147	A:	LEU	91	[	O	]	2.96	B:	TYR	83	[	N	]
148	A:	PHE	93	[	CE2	]	3.82	B:	TYR	83	[	CB	]
149	A:	LEU	57	[	CD1	]	3.74	B:	TYR	83	[	CD2	]
150	A:	LEU	91	[	CB	]	3.34	B:	TYR	83	[	O	]
151	A:	LEU	91	[	CD1	]	3.96	B:	TYR	83	[	O	]
152	A:	LEU	91	[	CG	]	3.61	B:	TYR	83	[	O	]
153	A:	VAL	90	[	C	]	4.00	B:	TYR	83	[	O	]
154	A:	LEU	91	[	CA	]	3.64	B:	TYR	83	[	O	]
155	A:	LEU	91	[	N	]	3.06	B:	TYR	83	[	O	]

156	A:	LEU	91	[	O	]	3.69	B:	TYR	83	[	O	]
157	A:	GLN	89	[	O	]	3.35	B:	ASN	84	[	CA	]
158	A:	GLN	89	[	O	]	3.99	B:	ASN	84	[	CB	]
159	A:	VAL	90	[	CG1	]	3.98	B:	ASN	84	[	CG	]
160	A:	VAL	90	[	CA	]	3.42	B:	ASN	84	[	OD1	]
161	A:	VAL	90	[	CB	]	3.73	B:	ASN	84	[	OD1	]
162	A:	VAL	90	[	C	]	3.70	B:	ASN	84	[	OD1	]
163	A:	VAL	90	[	CG1	]	3.34	B:	ASN	84	[	OD1	]
164	A:	LEU	91	[	N	]	3.07	B:	ASN	84	[	OD1	]
165	A:	LEU	91	[	O	]	3.52	B:	ASN	84	[	OD1	]
166	A:	GLN	89	[	O	]	3.59	B:	ASN	84	[	C	]
167	A:	GLN	89	[	O	]	2.89	B:	VAL	85	[	N	]
168	A:	GLN	89	[	O	]	3.94	B:	VAL	85	[	CA	]
169	A:	ASP	88	[	OD2	]	3.57	B:	VAL	85	[	C	]
170	A:	GLN	89	[	CA	]	3.90	B:	VAL	85	[	O	]
171	A:	GLN	89	[	O	]	3.49	B:	VAL	85	[	O	]
172	A:	ASP	88	[	CA	]	3.53	B:	VAL	85	[	O	]
173	A:	ASP	88	[	C	]	3.71	B:	VAL	85	[	O	]
174	A:	GLN	89	[	N	]	2.94	B:	VAL	85	[	O	]
175	A:	ASP	88	[	CG	]	3.73	B:	VAL	85	[	O	]
176	A:	ASP	88	[	OD2	]	3.32	B:	VAL	85	[	O	]
177	A:	ASP	88	[	OD2	]	3.42	B:	ARG	86	[	N	]
178	A:	VAL	87	[	O	]	3.68	B:	ARG	86	[	CA	]
179	A:	ASP	88	[	OD2	]	3.15	B:	ARG	86	[	CA	]
180	A:	ASP	88	[	OD2	]	3.23	B:	ARG	86	[	CB	]
181	A:	VAL	87	[	O	]	3.90	B:	ARG	86	[	CG	]
182	A:	VAL	87	[	O	]	3.65	B:	ARG	86	[	CD	]
183	A:	ASP	88	[	OD2	]	3.69	B:	ARG	86	[	CD	]
184	A:	VAL	87	[	O	]	3.69	B:	ARG	86	[	C	]
185	A:	VAL	87	[	O	]	2.84	B:	PHE	87	[	N	]
186	A:	VAL	87	[	C	]	3.87	B:	PHE	87	[	N	]
187	A:	VAL	87	[	O	]	3.69	B:	PHE	87	[	CA	]
188	A:	GLN	86	[	O	]	3.96	B:	PHE	87	[	CB	]
189	A:	VAL	87	[	O	]	3.75	B:	PHE	87	[	CB	]
190	A:	GLN	86	[	CD	]	3.82	B:	PHE	87	[	CE2	]
191	A:	GLN	86	[	O	]	3.58	B:	PHE	87	[	CD2	]
192	A:	VAL	87	[	O	]	3.71	B:	PHE	87	[	CD2	]
193	A:	VAL	87	[	C	]	3.72	B:	PHE	87	[	CD2	]
194	A:	ASP	88	[	N	]	3.84	B:	PHE	87	[	CD2	]
195	A:	VAL	87	[	O	]	3.52	B:	PHE	87	[	O	]
196	A:	ASP	88	[	OD2	]	3.47	B:	ARG	134	[	CZ	]
197	A:	ASP	88	[	OD1	]	3.28	B:	ARG	134	[	NH1	]
198	A:	ASP	88	[	CG	]	3.62	B:	ARG	134	[	NH1	]
199	A:	ASP	88	[	OD2	]	3.14	B:	ARG	134	[	NH1	]

200	A:	VAL	90	[	CG2	]	3.76	B:	ARG	134	[	NH1	]
201	A:	ASP	88	[	CG	]	3.98	B:	ARG	134	[	NH2	]
202	A:	ASP	88	[	OD2	]	2.98	B:	ARG	134	[	NH2	]

Supplementary Table 3 List of intermolecular interactions (≤ 4 Å) between two *Mtb*HadAB dimers in the tetramer.

Interactions are calculated by NCONTACT program of CCP4, Hydrogen bonds are highlighted in green, while salt bridges are highlighted in blue.

No.		x,y,z					Dist. [Å]		-y,-x,-z-1/2				
1	A:	ASP	16	[	CG	]	3.44	A:	ARG	22	[	NH2	]
2	A:	ASP	16	[	OD1	]	3.63	A:	ARG	22	[	NH1	]
3	A:	ASP	16	[	OD1	]	3.82	A:	ARG	22	[	CZ	]
4	A:	ASP	16	[	OD1	]	3.13	A:	ARG	22	[	NH2	]
5	A:	ASP	16	[	OD2	]	3.86	A:	ARG	22	[	NH1	]
6	A:	ASP	16	[	OD2	]	3.84	A:	ARG	22	[	CZ	]
7	A:	ASP	16	[	OD2	]	3.30	A:	ARG	22	[	NH2	]
8	A:	ASP	16	[	OD2	]	3.28	A:	ARG	26	[	CZ	]
9	A:	ASP	16	[	OD2	]	2.88	A:	ARG	26	[	NH2	]
10	A:	ASP	16	[	OD2	]	2.93	A:	ARG	26	[	NH1	]
11	A:	HIS	17	[	CG	]	3.70	A:	ARG	22	[	NH2	]
12	A:	HIS	17	[	ND1	]	3.67	A:	ARG	22	[	NH2	]
13	A:	HIS	17	[	CE1	]	3.40	A:	ARG	22	[	NH1	]
14	A:	HIS	17	[	CE1	]	3.54	A:	ARG	22	[	CZ	]
15	A:	HIS	17	[	CE1	]	3.47	A:	ARG	22	[	NH2	]
16	A:	HIS	17	[	NE2	]	3.68	A:	ARG	22	[	NH1	]
17	A:	HIS	17	[	NE2	]	3.89	A:	ARG	22	[	NE	]
18	A:	HIS	17	[	NE2	]	3.40	A:	ARG	22	[	CZ	]
19	A:	HIS	17	[	NE2	]	3.34	A:	ARG	22	[	NH2	]
20	A:	HIS	17	[	CD2	]	3.99	A:	ARG	22	[	CZ	]
21	A:	HIS	17	[	CD2	]	3.48	A:	ARG	22	[	NH2	]
22	A:	HIS	17	[	C	]	3.46	A:	ARG	22	[	NH2	]
23	A:	HIS	17	[	O	]	3.87	A:	ARG	22	[	CZ	]
24	A:	HIS	17	[	O	]	2.57	A:	ARG	22	[	NH2	]
25	A:	HIS	17	[	O	]	3.73	A:	GLU	23	[	OE2	]
26	A:	TYR	18	[	CA	]	3.51	A:	GLU	23	[	OE2	]
27	A:	TYR	18	[	CE2	]	3.82	A:	GLU	23	[	CG	]
28	A:	TYR	18	[	CD2	]	3.93	A:	GLU	23	[	CG	]
29	A:	TYR	18	[	CD2	]	3.82	A:	GLU	23	[	CD	]
30	A:	TYR	18	[	C	]	3.72	A:	GLU	23	[	OE2	]
31	A:	GLU	19	[	N	]	3.76	A:	GLU	23	[	CG	]

32	A:	GLU	19	[	N	]	2.96	A:	GLU	23	[	OE2	]
33	A:	GLU	19	[	N	]	3.73	A:	GLU	23	[	CD	]
34	A:	GLU	19	[	CA	]	3.97	A:	GLU	23	[	OE2	]
35	A:	GLU	19	[	CB	]	3.91	A:	GLU	23	[	OE2	]
36	A:	GLU	19	[	CG	]	3.90	A:	ARG	22	[	NE	]
37	A:	GLU	19	[	CD	]	3.80	A:	ARG	22	[	CD	]
38	A:	GLU	19	[	CD	]	3.81	A:	ARG	22	[	NE	]
39	A:	GLU	19	[	OE1	]	3.89	A:	ARG	22	[	CG	]
40	A:	GLU	19	[	OE2	]	3.77	A:	ARG	22	[	CD	]
41	A:	GLU	19	[	OE2	]	3.78	A:	ARG	22	[	NE	]
42	A:	GLU	19	[	O	]	3.35	A:	GLU	23	[	CG	]
43	A:	GLU	21	[	CA	]	3.68	A:	GLU	21	[	OE1	]
44	A:	GLU	21	[	CA	]	3.75	A:	GLU	21	[	CD	]
45	A:	GLU	21	[	CA	]	3.95	A:	GLU	21	[	OE2	]
46	A:	GLU	21	[	CB	]	3.19	A:	GLU	21	[	CG	]
47	A:	GLU	21	[	CB	]	3.03	A:	GLU	21	[	OE1	]
48	A:	GLU	21	[	CB	]	3.64	A:	GLU	21	[	CB	]
49	A:	GLU	21	[	CB	]	2.62	A:	GLU	21	[	CD	]
50	A:	GLU	21	[	CB	]	2.66	A:	GLU	21	[	OE2	]
51	A:	GLU	21	[	CG	]	3.20	A:	GLU	21	[	CG	]
52	A:	GLU	21	[	CG	]	3.98	A:	GLU	21	[	OE1	]
53	A:	GLU	21	[	CG	]	3.19	A:	GLU	21	[	CB	]
54	A:	GLU	21	[	CG	]	3.32	A:	GLU	21	[	CD	]
55	A:	GLU	21	[	CG	]	3.45	A:	GLU	21	[	OE2	]
56	A:	GLU	21	[	CD	]	3.75	A:	GLU	21	[	CA	]
57	A:	GLU	21	[	CD	]	3.32	A:	GLU	21	[	CG	]
58	A:	GLU	21	[	CD	]	2.62	A:	GLU	21	[	CB	]
59	A:	GLU	21	[	CD	]	3.96	A:	GLU	21	[	C	]
60	A:	GLU	21	[	CD	]	4.00	A:	ARG	22	[	N	]
61	A:	GLU	21	[	CD	]	3.70	A:	GLU	23	[	N	]
62	A:	GLU	21	[	CD	]	3.82	A:	GLU	23	[	CB	]
63	A:	GLU	21	[	CD	]	3.64	A:	GLU	21	[	CD	]
64	A:	GLU	21	[	CD	]	3.45	A:	GLU	21	[	OE2	]
65	A:	GLU	21	[	CD	]	3.92	A:	GLU	23	[	CG	]
66	A:	GLU	21	[	OE1	]	3.68	A:	GLU	21	[	CA	]
67	A:	GLU	21	[	OE1	]	3.98	A:	GLU	21	[	CG	]
68	A:	GLU	21	[	OE1	]	3.03	A:	GLU	21	[	CB	]
69	A:	GLU	21	[	OE1	]	3.53	A:	GLU	21	[	C	]
70	A:	GLU	21	[	OE1	]	3.18	A:	ARG	22	[	N	]
71	A:	GLU	21	[	OE1	]	3.95	A:	ARG	22	[	CA	]
72	A:	GLU	21	[	OE1	]	3.86	A:	ARG	22	[	C	]
73	A:	GLU	21	[	OE1	]	2.91	A:	GLU	23	[	N	]
74	A:	GLU	21	[	OE1	]	3.74	A:	GLU	23	[	CA	]
75	A:	GLU	21	[	OE1	]	3.47	A:	GLU	23	[	CB	]

76	A:	GLU	21	[	OE1	]	3.31	A:	GLU	23	[	CG	]
77	A:	GLU	21	[	OE2	]	3.95	A:	GLU	21	[	CA	]
78	A:	GLU	21	[	OE2	]	3.45	A:	GLU	21	[	CG	]
79	A:	GLU	21	[	OE2	]	2.66	A:	GLU	21	[	CB	]
80	A:	GLU	21	[	OE2	]	3.74	A:	GLU	23	[	N	]
81	A:	GLU	21	[	OE2	]	3.60	A:	GLU	23	[	CB	]
82	A:	GLU	21	[	OE2	]	3.49	A:	LYS	24	[	N	]
83	A:	GLU	21	[	OE2	]	3.45	A:	GLU	21	[	CD	]
84	A:	GLU	21	[	OE2	]	2.84	A:	GLU	21	[	OE2	]
85	A:	GLU	21	[	C	]	3.53	A:	GLU	21	[	OE1	]
86	A:	GLU	21	[	C	]	3.96	A:	GLU	21	[	CD	]
87	A:	ARG	22	[	N	]	3.18	A:	GLU	21	[	OE1	]
88	A:	ARG	22	[	N	]	4.00	A:	GLU	21	[	CD	]
89	A:	ARG	22	[	CA	]	3.95	A:	GLU	21	[	OE1	]
90	A:	ARG	22	[	CG	]	3.89	A:	GLU	19	[	OE1	]
91	A:	ARG	22	[	CD	]	3.77	A:	GLU	19	[	OE2	]
92	A:	ARG	22	[	CD	]	3.80	A:	GLU	19	[	CD	]
93	A:	ARG	22	[	NE	]	3.89	A:	HIS	17	[	NE2	]
94	A:	ARG	22	[	NE	]	3.78	A:	GLU	19	[	OE2	]
95	A:	ARG	22	[	NE	]	3.90	A:	GLU	19	[	CG	]
96	A:	ARG	22	[	NE	]	3.81	A:	GLU	19	[	CD	]
97	A:	ARG	22	[	CZ	]	3.87	A:	HIS	17	[	O	]
98	A:	ARG	22	[	CZ	]	3.82	A:	ASP	16	[	OD1	]
99	A:	ARG	22	[	CZ	]	3.84	A:	ASP	16	[	OD2	]
100	A:	ARG	22	[	CZ	]	3.40	A:	HIS	17	[	NE2	]
101	A:	ARG	22	[	CZ	]	3.99	A:	HIS	17	[	CD2	]
102	A:	ARG	22	[	CZ	]	3.54	A:	HIS	17	[	CE1	]
103	A:	ARG	22	[	NH1	]	3.63	A:	ASP	16	[	OD1	]
104	A:	ARG	22	[	NH1	]	3.86	A:	ASP	16	[	OD2	]
105	A:	ARG	22	[	NH1	]	3.68	A:	HIS	17	[	NE2	]
106	A:	ARG	22	[	NH1	]	3.40	A:	HIS	17	[	CE1	]
107	A:	ARG	22	[	NH2	]	3.46	A:	HIS	17	[	C	]
108	A:	ARG	22	[	NH2	]	2.57	A:	HIS	17	[	O	]
109	A:	ARG	22	[	NH2	]	3.44	A:	ASP	16	[	CG	]
110	A:	ARG	22	[	NH2	]	3.13	A:	ASP	16	[	OD1	]
111	A:	ARG	22	[	NH2	]	3.30	A:	ASP	16	[	OD2	]
112	A:	ARG	22	[	NH2	]	3.70	A:	HIS	17	[	CG	]
113	A:	ARG	22	[	NH2	]	3.34	A:	HIS	17	[	NE2	]
114	A:	ARG	22	[	NH2	]	3.48	A:	HIS	17	[	CD2	]
115	A:	ARG	22	[	NH2	]	3.67	A:	HIS	17	[	ND1	]
116	A:	ARG	22	[	NH2	]	3.47	A:	HIS	17	[	CE1	]
117	A:	ARG	22	[	C	]	3.86	A:	GLU	21	[	OE1	]
118	A:	GLU	23	[	N	]	2.91	A:	GLU	21	[	OE1	]
119	A:	GLU	23	[	N	]	3.70	A:	GLU	21	[	CD	]

120	A:	GLU	23	[	N	]	3.74	A:	GLU	21	[	OE2	]
121	A:	GLU	23	[	CA	]	3.74	A:	GLU	21	[	OE1	]
122	A:	GLU	23	[	CB	]	3.47	A:	GLU	21	[	OE1	]
123	A:	GLU	23	[	CB	]	3.82	A:	GLU	21	[	CD	]
124	A:	GLU	23	[	CB	]	3.60	A:	GLU	21	[	OE2	]
125	A:	GLU	23	[	CG	]	3.82	A:	TYR	18	[	CE2	]
126	A:	GLU	23	[	CG	]	3.93	A:	TYR	18	[	CD2	]
127	A:	GLU	23	[	CG	]	3.76	A:	GLU	19	[	N	]
128	A:	GLU	23	[	CG	]	3.35	A:	GLU	19	[	O	]
129	A:	GLU	23	[	CG	]	3.31	A:	GLU	21	[	OE1	]
130	A:	GLU	23	[	CG	]	3.92	A:	GLU	21	[	CD	]
131	A:	GLU	23	[	CD	]	3.82	A:	TYR	18	[	CD2	]
132	A:	GLU	23	[	CD	]	3.73	A:	GLU	19	[	N	]
133	A:	GLU	23	[	OE2	]	3.73	A:	HIS	17	[	O	]
134	A:	GLU	23	[	OE2	]	3.51	A:	TYR	18	[	CA	]
135	A:	GLU	23	[	OE2	]	3.72	A:	TYR	18	[	C	]
136	A:	GLU	23	[	OE2	]	2.96	A:	GLU	19	[	N	]
137	A:	GLU	23	[	OE2	]	3.97	A:	GLU	19	[	CA	]
138	A:	GLU	23	[	OE2	]	3.91	A:	GLU	19	[	CB	]
139	A:	LYS	24	[	N	]	3.49	A:	GLU	21	[	OE2	]
140	A:	LYS	24	[	CE	]	3.49	A:	GLU	27	[	OE1	]
141	A:	LYS	24	[	NZ	]	3.39	A:	GLU	27	[	CD	]
142	A:	LYS	24	[	NZ	]	2.74	A:	GLU	27	[	OE1	]
143	A:	LYS	24	[	NZ	]	3.35	A:	GLU	27	[	OE2	]
144	A:	ARG	26	[	CZ	]	3.28	A:	ASP	16	[	OD2	]
145	A:	ARG	26	[	NH1	]	2.93	A:	ASP	16	[	OD2	]
146	A:	ARG	26	[	NH2	]	2.88	A:	ASP	16	[	OD2	]
147	A:	GLU	27	[	CD	]	3.39	A:	LYS	24	[	NZ	]
148	A:	GLU	27	[	OE1	]	3.49	A:	LYS	24	[	CE	]
149	A:	GLU	27	[	OE1	]	2.74	A:	LYS	24	[	NZ	]
150	A:	GLU	27	[	OE2	]	3.35	A:	LYS	24	[	NZ	]
151	A:	GLU	27	[	OE2	]	3.73	A:	GLU	27	[	OE2	]
152	B:	ARG	24	[	CG	]	3.70	B:	TRP	42	[	CZ3	]
153	B:	GLN	25	[	CA	]	3.94	B:	TRP	42	[	CH2	]
154	B:	GLN	25	[	CA	]	3.96	B:	TRP	42	[	CZ2	]
155	B:	GLN	25	[	CB	]	3.99	B:	TRP	42	[	CZ2	]
156	B:	GLN	25	[	CD	]	3.87	B:	LEU	37	[	CB	]
157	B:	GLN	25	[	OE1	]	3.80	B:	LEU	37	[	CB	]
158	B:	GLN	25	[	OE1	]	3.37	B:	LEU	37	[	O	]
159	B:	VAL	28	[	CG1	]	3.96	B:	TRP	42	[	CD1	]
160	B:	VAL	28	[	CG1	]	3.99	B:	TRP	42	[	NE1	]
161	B:	VAL	28	[	CG2	]	3.86	B:	TRP	42	[	CD2	]
162	B:	VAL	28	[	CG2	]	3.75	B:	TRP	42	[	CE3	]
163	B:	ASN	29	[	CB	]	3.80	B:	LEU	37	[	CD2	]

164	B:	ASN	29	[	ND2	]	3.79	B:	LEU	37	[	CD1	]
165	B:	ASN	29	[	ND2	]	3.72	B:	GLY	32	[	CA	]
166	B:	ASN	29	[	ND2	]	3.44	B:	LEU	37	[	CD2	]
167	B:	ASN	29	[	O	]	3.73	B:	GLY	32	[	CA	]
168	B:	GLY	32	[	CA	]	3.73	B:	ASN	29	[	O	]
169	B:	GLY	32	[	CA	]	3.72	B:	ASN	29	[	ND2	]
170	B:	GLY	32	[	O	]	3.59	B:	VAL	33	[	CG2	]
171	B:	VAL	33	[	CG2	]	3.59	B:	GLY	32	[	O	]
172	B:	LEU	37	[	CB	]	3.87	B:	GLN	25	[	CD	]
173	B:	LEU	37	[	CB	]	3.80	B:	GLN	25	[	OE1	]
174	B:	LEU	37	[	CD1	]	3.79	B:	ASN	29	[	ND2	]
175	B:	LEU	37	[	CD2	]	3.80	B:	ASN	29	[	CB	]
176	B:	LEU	37	[	CD2	]	3.44	B:	ASN	29	[	ND2	]
177	B:	LEU	37	[	O	]	3.37	B:	GLN	25	[	OE1	]
178	B:	TRP	42	[	CD1	]	3.96	B:	VAL	28	[	CG1	]
179	B:	TRP	42	[	NE1	]	3.99	B:	VAL	28	[	CG1	]
180	B:	TRP	42	[	CD2	]	3.86	B:	VAL	28	[	CG2	]
181	B:	TRP	42	[	CE3	]	3.75	B:	VAL	28	[	CG2	]
182	B:	TRP	42	[	CZ3	]	3.70	B:	ARG	24	[	CG	]
183	B:	TRP	42	[	CH2	]	3.94	B:	GLN	25	[	CA	]
184	B:	TRP	42	[	CZ2	]	3.96	B:	GLN	25	[	CA	]
185	B:	TRP	42	[	CZ2	]	3.99	B:	GLN	25	[	CB	]
186	B:	ASP	43	[	CG	]	3.60	B:	ASN	95	[	ND2	]
187	B:	ASP	43	[	OD2	]	3.68	B:	ASN	95	[	CG	]
188	B:	ASP	43	[	OD2	]	3.75	B:	ASN	95	[	OD1	]
189	B:	ASP	43	[	OD2	]	2.80	B:	ASN	95	[	ND2	]
190	B:	ASN	95	[	CG	]	3.68	B:	ASP	43	[	OD2	]
191	B:	ASN	95	[	OD1	]	3.75	B:	ASP	43	[	OD2	]
192	B:	ASN	95	[	ND2	]	3.60	B:	ASP	43	[	CG	]
193	B:	ASN	95	[	ND2	]	2.80	B:	ASP	43	[	OD2	]
